# Developmental cannabidiol exposure increases anxiety and modifies genome-wide brain DNA methylation in adult female mice

**DOI:** 10.1186/s13148-020-00993-4

**Published:** 2021-01-06

**Authors:** Nicole M. Wanner, Mathia Colwell, Chelsea Drown, Christopher Faulk

**Affiliations:** 1 Department of Veterinary and Biomedical Sciences, University of Minnesota, 1334 Eckles Avenue, St. Paul, MN USA; 2 Department of Animal Science, University of Minnesota, 1334 Eckles Avenue, 225 Food Science, St. Paul, MN 55018 USA

**Keywords:** Cannabis, Anxiety, Memory, Prenatal, Epigenetics, Addiction, DOHaD

## Abstract

**Background:**

Use of cannabidiol (CBD), the primary non-psychoactive compound found in cannabis, has recently risen dramatically, while relatively little is known about the underlying molecular mechanisms of its effects. Previous work indicates that direct CBD exposure strongly impacts the brain, with anxiolytic, antidepressant, antipsychotic, and other effects being observed in animal and human studies. The epigenome, particularly DNA methylation, is responsive to environmental input and can direct persistent patterns of gene regulation impacting phenotype. Epigenetic perturbation is particularly impactful during embryogenesis, when exogenous exposures can disrupt critical resetting of epigenetic marks and impart phenotypic effects lasting into adulthood. The impact of prenatal CBD exposure has not been evaluated; however, studies using the psychomimetic cannabinoid Δ9-tetrahydrocannabinol (THC) have identified detrimental effects on psychological outcomes in developmentally exposed adult offspring. We hypothesized that developmental CBD exposure would have similar negative effects on behavior mediated in part by the epigenome. Nulliparous female wild-type Agouti viable yellow (*A*^vy^) mice were exposed to 20 mg/kg CBD or vehicle daily from two weeks prior to mating through gestation and lactation. Coat color shifts, a readout of DNA methylation at the Agouti locus in this strain, were measured in F1 *A*^*vy*^*/a* offspring. Young adult F1 *a/a* offspring were then subjected to tests of working spatial memory and anxiety/compulsive behavior. Reduced-representation bisulfite sequencing was performed on both F0 and F1 cerebral cortex and F1 hippocampus to identify genome-wide changes in DNA methylation for direct and developmental exposure, respectively.

**Results:**

F1 offspring exposed to CBD during development exhibited increased anxiety and improved memory behavior in a sex-specific manner. Further, while no significant coat color shift was observed in *A*^*vy*^*/a* offspring, thousands of differentially methylated loci (DMLs) were identified in both brain regions with functional enrichment for neurogenesis, substance use phenotypes, and other psychologically relevant terms.

**Conclusions:**

These findings demonstrate for the first time that despite positive effects of direct exposure, developmental CBD is associated with mixed behavioral outcomes and perturbation of the brain epigenome.

## Background

Cannabidiol (CBD) is the primary non-psychomimetic compound found in cannabis (*Cannabis sativa*) and an FDA-approved treatment for childhood epilepsy that also shows therapeutic potential for several neuropsychiatric disorders. Use of both cannabis and CBD is rising in the USA with CBD sales expected to reach 1.8 billion dollars by 2022 [1], due in part to reports of their positive effects on psychological phenotypes. In rodent studies, exposure to CBD in adulthood has been shown to reduce immobility and increase swimming time in the forced swim test, a measure of depression [2, 3], increase time spent in the open arm of the elevated plus maze, a measure of anxiety [4–7], and reduce responsiveness to drugs of addiction such as morphine and cocaine [4, 8]. In human trials, CBD additionally reduces psychotic symptoms in schizophrenia [9, 10] and lowers subjective measures of anxiety [11, 12]. These findings contrast with previous research on the psychoactive cannabinoid Δ9-tetrahydrocannabinol (THC), which report anxiogenic and other adverse psychological effects that concurrent CBD administration may counteract [13–15]. In vitro and in vivo studies suggest that the underlying mechanisms behind CBD’s actions in the brain are complex. CBD acts on a large number of targets including serotonin 1a (*5HT-1a*), peroxisome proliferator-activated receptor gamma (*PPARγ*), and transient receptor potential cation channel subfamily V (*TRPV*) receptors, antioxidant mechanisms, and modulation of endocannabinoid levels [5, 7, 16–19]. CBD also appears to impact neurogenesis in the hippocampal dentate gyrus, one of only two sites of ongoing neurogenesis in the adult brain. Luján, Cantacorps, and Valverde demonstrated that the protective effects of CBD on voluntary cocaine intake were ameliorated by pharmacological inhibition of hippocampal neurogenesis, and Campos et al. determined that CBD’s anxiolytic effects were driven by facilitating endocannabinoid-mediated neurogenesis using a mouse model of chronic unpredictable stress [16, 20].

Despite the potential for therapeutic applications of direct CBD exposure, its widespread actions in the brain and increasing use of cannabinoids during pregnancy raise concern for potential impacts on the developing fetus and subsequent adult. Administration of THC during pregnancy has been associated with negative cognitive outcomes in rodent models [21]. Notable examples include a study by de Salas-Quiroga et al., who identified sex-specific deficits in spatial memory in mice prenatally exposed to THC [22, 23], and work by Trezza et al. associating perinatal THC with altered vocalization and social and play behavior in rats [24]. Mereu et al. similarly found that the synthetic cannabinoid CB1 receptor agonist WIN 55212-2, which mimics the actions of THC, disrupted memory retention and led to hyperactive behavior in prenatally exposed adult rats [25]. Effects on addiction-related behavior have also been an area of interest for cannabinoid exposure during pregnancy, with reports of increased heroin seeking [26] and morphine self-administration [27] and modified dopamine [28] and enkephalin [29] signaling being reported. Perturbed glutamatergic [30], GABAergic [31], and serotonergic signaling [32] have also been observed, reflecting the widespread impact of exogenous cannabinoids in the brain.

The developmental origins of health and disease (DOHaD) hypothesis state that early-life environmental exposures can mediate later life phenotypes via epigenomic perturbation [33–36]. DNA methylation is the most commonly studied epigenetic mark and occurs when a methyl group is added to the fifth carbon of cytosine in a cytosine-guanine dinucleotide (CpG) context in mammals. Differential methylation is highly relevant for neuropsychiatric diseases and has been identified in association with schizophrenia [37–42], depression [43–46], anxiety [47–50], and autism spectrum disorder [51–55]. Several studies have identified differential methylation patterns in the sperm of humans and rats directly exposed to THC, particularly at the autism candidate locus *DLGAP2*, and a study by Watson et al. demonstrated that parental THC shifts DNA methylation of genes relevant for glutamatergic signaling in the rat nucleus accumbens [56–58]. Importantly, the effects of parental exposure to CBD have not yet been explored. The presence of cannabinoids during critical windows of methylation pattern setting in development has the potential to persistently alter patterns of gene regulation in the brain; these changes are likely to contribute to adverse neuropsychiatric phenotypes in adulthood.

In this context, the aim of the present study was to investigate the effects of developmental exposure to CBD on adult behavior and the brain methylome. To this end, we subjected pregnant mice to a subchronic CBD exposure paradigm and tested their abstinent adult offspring for abnormalities in memory and anxiety behavior. Regarding DNA methylation, we utilized the Agouti viable yellow (*A*^*vy*^) environmental biosensor model [59], which provides a readout of methylation changes at the *Agouti* locus via offspring coat color and has been successfully used to identify epigenomic perturbations associated with prenatal exposure to bisphenol A [60], lead [61], and other compounds. For a genome-wide perspective on a target tissue for neuropsychiatric phenotypes, we performed reduced-representation bisulfite sequencing (RRBS) in the cerebral cortex and hippocampus of adult F1 offspring. We found that developmental CBD exposure resulted in a sex-specific increase in anxiety behavior affecting female offspring and identified over 2000 differentially methylated loci in each brain region. Thousands of differentially methylated loci were additionally identified in the cortex of directly exposed F0 females in the absence of anxiety or memory changes, echoing recent studies showing modified methylation with CBD exposure and recapitulating behavior studies demonstrating a lack of effect in the absence of a stressor. Overall, these findings provide an initial investigation into the effects of prenatal exposure to CBD, identify behavior deficits and functionally relevant methylation changes in the brain, and support the importance of neuroepigenetics in the etiology of psychiatric phenotypes.

## Methods

### Animals

Animals were maintained in accordance with the Guidelines for the Care and Use of Laboratory Animals and were treated humanely and with regard for alleviation of suffering. The study protocol was approved by the University of Minnesota Institutional Animal Care and Use Committee (IACUC). Mice were obtained from an Agouti viable yellow (*A*^*vy*^) colony maintained for over 220 generations with the *A*^*vy*^ allele passed through the male line, resulting in forced heterozygosity on a genetically invariant background with 93% identity to C57BL/6 [62, 63]. All animals were maintained on a standard chow diet (Envigo Teklad 19% protein 2019 breeder diet for dams and 18% protein 2018 maintenance diet for offspring) and housed in cages of 3–4 individuals on corn cob bedding with a 12-h light/dark cycle.

### Exposure paradigm

Pharmaceutical-grade CBD (Epidiolex, GW Pharmaceuticals, Cambridge, UK) was purchased at the University of Minnesota Boynton Health Pharmacy (Minneapolis, MN). CBD was diluted to 10 mg/mL concentration in honey (Nice! Organic Honey, Walgreens) due to its high lipophilicity and stability at room temperature. Honey has been used successfully as a drug delivery vehicle by others [64]. Twenty-two six- to ten-week-old, sexually mature nulliparous wild-type *a/a* females were randomized into two groups and received either 20 mg/kg Epidiolex™ (GW Pharmaceuticals, Cambridge, UK) emulsified in honey or vehicle only daily via oral administration using the tip of a 14-gauge gavage needle for 14 days prior to mating. This dose was chosen based on previous CBD studies and approximates casual human use (~ 1.5 mg/kg) due to scaling factors for body surface area [3, 4, 8, 65, 66]. On day 14, F0 females were harem-mated with *A*^*vy*^*/a* males (8–12 weeks of age) and daily dosing continued through gestation, lactation, and behavior testing for a total exposure time of approximately 9 weeks. All animals had access to food and drinking water ad libitum throughout the experiment in accordance with the Institute of Laboratory Animal Resources guidelines [67]. F1 animals were drug-abstinent following weaning.

### Behavior procedures

F0 exposed and control dams were subjected to the Y-maze spontaneous alternation test (spatial working memory) and the marble burying task (anxiety and compulsive behavior) between 4–7 days following the weaning of pups with CBD exposure ongoing. Tests were conducted in the home mouse room during the light phase of the light–dark cycle. Each F0 female was tested twice in order to differentiate acute from cumulative CBD effects with consecutive tests being at least 24 h apart. For acute testing, dams were dosed with either 20 mg/kg CBD or vehicle between 0.5 and 1.5 h prior to testing to ensure CBD plasma levels were near *C*_max_ [68]. For cumulative effect testing, dams were tested approximately 24 h after the last dose to ensure CBD plasma levels were low. Adult *a/a* F1 offspring, which were drug-abstinent after weaning, were subjected to the same behavior tests once per animal at 12 weeks of age with at least 24 h between tests. Same sizes were as follows: F0, *n* = 9 control, 7 exposed; F1, *n* = 17 control, 16 exposed. F0 behavior testing was conducted on females only as males were not exposed to CBD, while F1 behavior testing was conducted on both males and females.

### Y-maze spontaneous alternation

The Y-maze spontaneous alternation task is a measure of spatial working memory and exploits rodents’ natural tendency to explore novel areas [69, 70]. Mice were placed at the end of one arm facing the center in a standard Y-maze (MazeEngineers, Boston, MA) consisting of a high-walled chamber with three arms connected at 120°. Investigators vacated the room, and the animal’s movement was recorded on video using a tripod and digital camera for ten minutes. After ten minutes, the animal was returned to the home cage and the apparatus was sanitized with 70% alcohol to prevent scent trails from confounding subsequent runs. The sequence of entries (all four feet within the arm) was recorded from the video by an investigator blinded to treatment group, and the spontaneous alternation percentage was calculated as the number of spontaneous alternations ÷ (number of entries—2) × 100. One spontaneous alternation was counted when three consecutive entries into unique arms (e.g., A, B, C) were recorded. The total number of arm entries was also recorded as a measure of exploration.

### Marble burying

The marble burying task is a measure of anxiety and compulsive behavior in mice and takes advantage of rodents’ natural tendency to bury objects [71–73]. Mice were individually placed into the corner of a rat cage filled with 10 cm (height) of corn cob bedding and 20 evenly placed marbles (4 × 5 layout) distributed on its surface. Testing was conducted with the investigator absent from the room for 30 min. After 30 min, mice were carefully removed from the test cage to avoid disturbing the bedding and the number of marbles buried was counted, with marbles at least 2/3 covered being counted as buried. The bedding was thoroughly mixed, and marbles were washed with dish soap and rinsed with 70% ethanol following each trial to prevent scent from affecting subsequent runs.

### *A*^*vy*^/a coat color

The *A*^*vy*^ strain was used to determine whether CBD exposure imparted large-scale changes in DNA methylation in developmentally exposed offspring. Briefly, the *Agouti* gene produces a paracrine signaling molecule that determines coat color, among other functions. The upstream region of the *Agouti* gene of *A*^*vy*^*/a* mice contains an intracisternal A particle (IAP) retrotransposon insertion that leads to constitutive, metastable expression of the gene with the magnitude of expression dependent upon stochastic DNA methylation within the insertion [74]. Shifts in methylation of these metastable loci can be triggered by in utero environmental exposures such as bisphenol A, resulting in a shifted distribution of coat colors in the affected offspring via variable production of pheomelanin [60]. Constitutive expression of the *Agouti* gene is not limited to hair follicles and thus leads to obesity, liver tumors, diabetes, and other phenotypes in *A*^*vy*^*/a* animals. Therefore, to eliminate confounding effects, only wild-type *a/a* animals produced by *A*^*vy*^*/a *× *a/a* breedings were used for molecular and behavioral analyses. *A*^*vy*^*/a* F1 offspring were photographed from above at 5–7 weeks of age in order to measure coat color, a readout of DNA methylation at the metastable *Agouti* epiallele in this strain. Coat color photographs were scored on a three-category scale (low brown mottling/low methylation, medium mottling/medium methylation, high mottling or pseudoagouti/high methylation) by two investigators blinded to treatment group. Discrepant scores were settled by a third blinded investigator to identify overall and sex-specific differences in methylation between CBD and control groups.

### Statistical analysis

Normality for behavior scores and coat color data were assessed using density plots and QQ plots. Between-group differences in F0 and F1 behavior scores (performed separately for both F0 dosage timing windows) were assessed using Wilcoxon rank-sum tests in RStudio. Two F1 female outliers (one control, one exposed) lying more than two standard deviations above the group mean were identified for Y-maze spontaneous alternation and removed from the analysis. Sex:group interactions for behavior tests were assessed using a one-way analysis of variance (ANOVA). Between-group differences for F0 acute and cumulative behavior scores were assessed using Wilcoxon rank-sum tests, and paired within-group scores (acute vs. cumulative) were assessed using Wilcoxon signed-rank tests. Chi-square tests for trend were used to evaluate overall and sex-specific differences in F1 *A*^*vy*^ coat color between groups. Wild-type F1 weights were measured from weaning through behavior testing, and differences between groups were assessed using ANOVA, while differences in litter size were determined using Wilcoxon rank-sum tests.

### DNA isolation and bisulfite sequencing

Samples were prepared, and sequencing was performed as described previously [75]. Briefly, animals were euthanized via isoflurane inhalation followed immediately by internal decapitation. The brain was removed and dissected fresh using a stereoscope to obtain cortical (F0 *n* = 6, F1 *n* = 6) and hippocampal (F1 *n* = 4) regions [76]. Tissue samples were placed directly in RNAlater (Sigma-Aldrich) and stored at 4 °C overnight, then transferred to -80 °C for long-term storage. Total genomic DNA (gDNA) was isolated from each animal using the DNeasy Blood and Tissue kit following the manufacturer’s protocol (Qiagen, Hilden, Germany). A NanoPhotometer N50 system was used to check DNA yield with three biological replicates per group being chosen for further processing based on concentration and quality. Methylation analyses were performed on F0 dams and F1 females only. gDNA was bisulfite-converted following isolation using the EZ DNA Methylation-Lightning Kit (Zymo Research, Irvine, CA). Bisulfite conversion allows detection of methylated cytosines via treatment of DNA with sodium bisulfite, which causes unmethylated cytosines to be deaminated to uracils. These loci are read as thymidine by polymerases during sequencing. Genome-wide DNA methylation levels were measured using reduced-representation bisulfite sequencing (RRBS) at Diagenode, S.A. (Belgium). Briefly, DNA concentration of samples was measured using the Qubit® dsDNA BR Assay Kit (ThermoFisher Scientific), and DNA quality was assessed using the Fragment Analyzer™ and DNF-488 High Sensitivity genomic DNA Analysis Kit (Agilent). RRBS libraries were prepared using the Premium Reduced Representation Bisulfite Sequencing Kit (Diagenode Cat# C02030033), and 100 ng of genomic DNA was used to start library preparation for each sample. Bisulfite sequencing was performed in single-end mode 50 bp (SE50) on an Illumina HiSeq 3000/4000. Quality control of reads was performed using FastQC version 0.11.8 [77], and adapter removal was performed using Trim Galore! Version 0.4.1 [78]. Bismark, a specialized tool that utilizes an in silico bisulfite-converted reference genome, was used for mapping bisulfite-treated reads [79]. The cytosine2coverage module of Bismark was used to determine the methylation state of all cytosines for every uniquely mappable read, determine their sequence context, and compute the percentage methylation. Spike-in control sequences were used to check the bisulfite conversion rates and to validate the efficiency of bisulfite treatment. The resulting cytosine loci were filtered to exclude non-CG context cytosines, loci with less than 10 reads, and loci with less than two biological replicates per group using R version 3.6.1.

### DML and DMR calling, annotation, and functional enrichment

RStudio open-source software (version 3.6.1) tools were used for RRBS analysis as described previously [75]. Briefly, the *DSS* R package (version 2.32.0) was used to test RRBS data for differential methylation between CBD-exposed and control animals [80]. The DMLtest, callDML, and callDMR functions in *DSS* were used to identify differentially methylated CpG loci (DMLs) and regions (DMRs) with *Δ* > 0.1 and local FDR < 0.001. The *annotatr* R package (version 1.10.0) was used to annotate DMLs and DMRs to the mm10 genome [81]. Predicted genes and three large erroneous gene transcripts (ENSMUST00000127664.1, ENSMUST00000124096.7, and ENSMUST00000154148.7), which were present in *annotatr*’s GENCODE-based intervals but not in RefSeq when assessed using UCSC Genome Browser, were manually removed from the annotation. The randomize_regions function and Chi-square tests were used to compare the observed genic distribution of DMLs to the expected distribution, and the plot_annotations function was used to generate figures. Functional enrichment of DML-containing genes was performed using the ToppGene suite tool ToppFun [82]. ToppFun utilizes hypergeometric distributions with Bonferroni correction to determine statistically significant enrichment in up to fourteen functional categories including Gene Ontology (GO) terms, human and mouse phenotypes, protein–protein interactions, diseases, and others [82]. Disease annotations are drawn from DisGeNET, Online Mendelian Inheritance in Man (OMIM) MedGen, and other sources. Lists of unique DML-containing genes for each assayed tissue (F1 cortex, F1 hippocampus, F0 cortex) were used as input selecting the “HGNC Symbol and Synonyms” entry type and run on default settings; terms with a Bonferroni-corrected p-value less than 0.05 were deemed significant.

### Pyrosequencing

Pyrosequencing was performed as described previously [83]. Briefly, LINE1 and intracisternal A particle (IAP) retrotransposon pyrosequencing primers were designed using Qiagen Pyromark Assay Design software version 2.0.2 and sequences from the mm10 genome. The parameters for each reaction included a thermocycler protocol of 95 °C for 30 s, an optimized temperature for 30 s, and 72 °C for 30 s repeated for 35–40 cycles. Primer sequences and conditions are presented in Additional file 1. DNA methylation level was quantitated from PCR products on a Qiagen Pyromark Q96 ID instrument. Controls consisted of a “no template control” and two wells of bisulfite converted 100% or 0% methylated control mouse DNA from EpiGentek. Methylation results were valued under criteria that the Pyromark software defined as ‘check’ or ‘passing’, with these values retained for analysis, and discarded if ‘failed’.

## Results

### Effects of developmental and direct CBD exposure on memory and anxiety

**Fig. 1:**
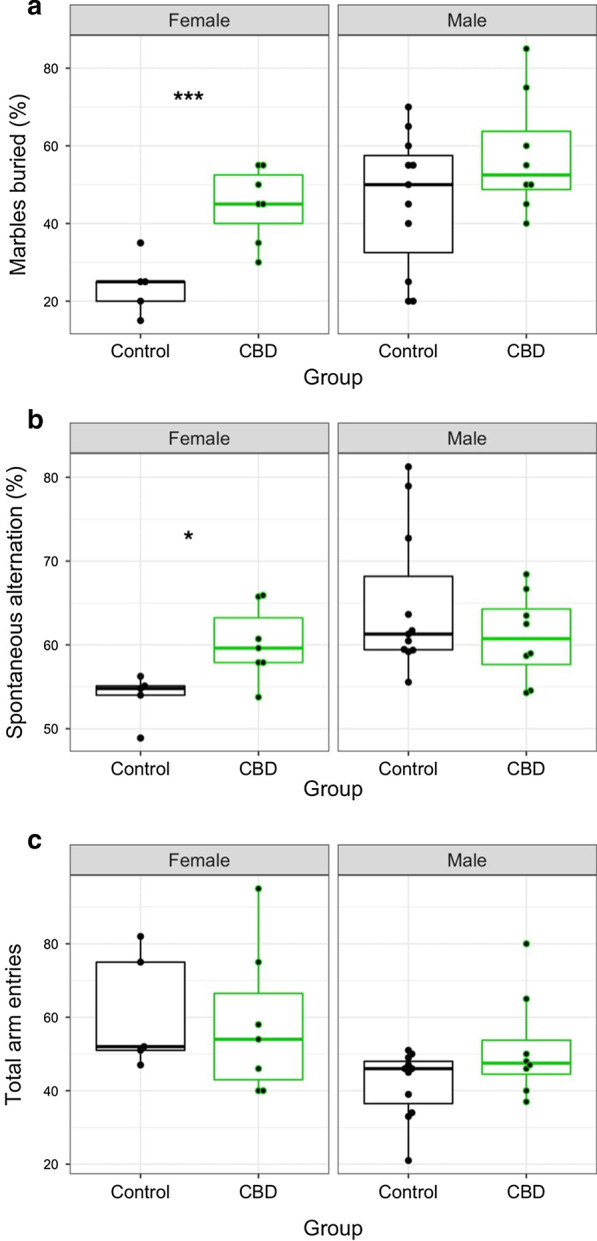
F1 behavior results stratified by sex. **a** Percent of marbles buried in the marble burying test, a measure of anxiety and compulsive behavior, in adult wild-type *a/a* offspring exposed to CBD or vehicle only during development. **b** Y-maze spontaneous alternation percentage scores, a measure of working spatial memory, for the same cohort of mice. **c** Y-maze total arm entries, a measure of locomotor activity. Each point represents an individual animal. *p < 0.05; ***p < 0.001

No significant differences in F1 weight from weaning through study conclusion (12 weeks) were identified by ANOVA (Additional file 2). CBD-exposed litters contained 1.25 more pups on average when compared to control litters (*p* = 0.0134; Additional file 3). To evaluate behavioral effects associated with developmental CBD exposure, F1 offspring of both sexes were subjected to the marble burying test, a measure of anxiety and compulsive behavior, and the Y-maze spontaneous alternation test, a measure of working spatial memory. No significant differences in marble burying or Y-maze spontaneous alternation were identified between the full control and CBD-exposed F1 groups (Additional file 4); however, significant sex interactions were identified by ANOVA for both behavior tests warranting stratification by sex. A sex effect regardless of treatment group was identified by ANOVA for marble burying (*p* = 0.00139), and both a sex effect (*p* = 0.0239) and a sex:treatment interaction (*p* = 0.0385) were identified for Y-maze spontaneous alternation. Stratifying results by sex revealed that young adult female F1 offspring exposed to CBD during gestation and preweaning buried nearly twice as many marbles as unexposed female controls (Fig. 1; *p* = 0.000328) indicating an increase in anxiety behavior, while differences between control and CBD-exposed F1 males were not significant (*p* = 0.156). Y-maze spontaneous alternation percentage, a measure of working spatial memory, was also increased in exposed adult female offspring (*p* = 0.0344). The total number of Y-maze arm entries, a measure of locomotor activity, was not significantly different between F1 groups of either sex.

Based on previous studies demonstrating anxiolytic effects for CBD and memory deficits for THC, F0 female behavior scores were also assessed during continuing CBD exposure following weaning of pups, representing approximately nine weeks of daily exposure. F0 behavior tests were conducted twice, once during CBD’s C_max_ approximately one hour after dosing and again at least 24 h after dosing to delineate acute and cumulative effects. F0 Y-maze spontaneous alternation percentages were not significantly different between control and exposed groups at either timing of dosage (Additional file 5; acute *p* = 0.114; cumulative *p* = 0.791). Similarly, differences in the total number of arm entries between F0 groups were not significantly different (acute *p* = 0.7449; cumulative *p* = 0.1004). F0 marble burying scores were also not significantly different between groups for either dosage window (acute *p* = 0.524; cumulative *p* = 0.421) or between dosage windows.

### Effects of CBD on DNA methylation

In order to assess the effects of developmental CBD exposure on DNA methylation, we used the Agouti viable yellow (*A*^*vy*^) environmental biosensor model. Coat color was visually assessed on a three-category scale in *A*^*vy*^*/a* F1 offspring of both sexes (*n* = 76). Changes in coat color distribution in this strain represent shifts in DNA methylation at the *Agouti* locus. Chi-square tests for trend revealed that differences in coat color distribution between control and exposed F1 *A*^*vy*^*/a* animals did not reach significance overall (*p* = 0.204) or within males (*p* = 0.874) or females (*p* = 0.0924) (Additional file 6).

**Fig. 2:**
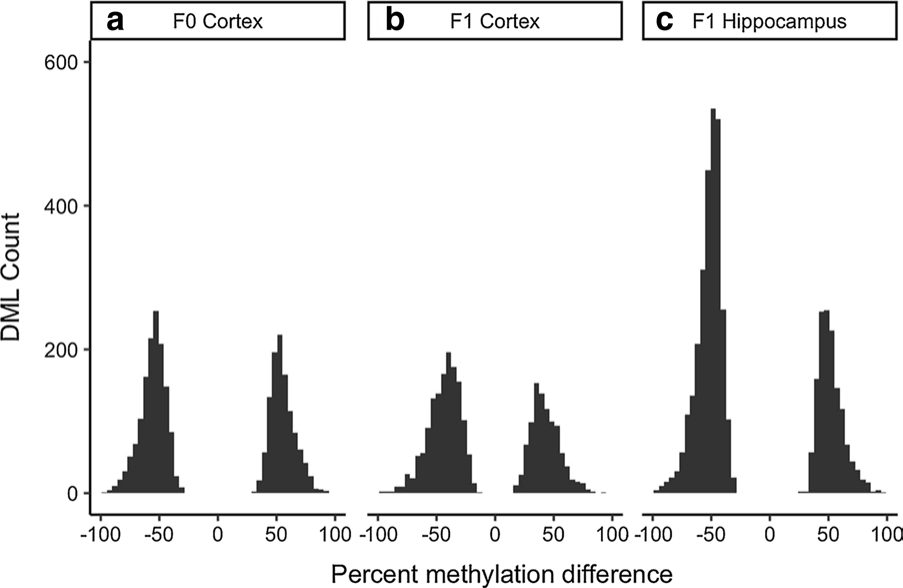
Genome-wide differences in brain methylation. Percentage difference (CBD exposed—control) at all statistically significant differentially methylated loci (DMLs) in **a** directly exposed F0 female cerebral cortex, **b** developmentally exposed F1 female cerebral cortex, and **c** developmentally exposed F1 female hippocampus

**Fig. 3:**
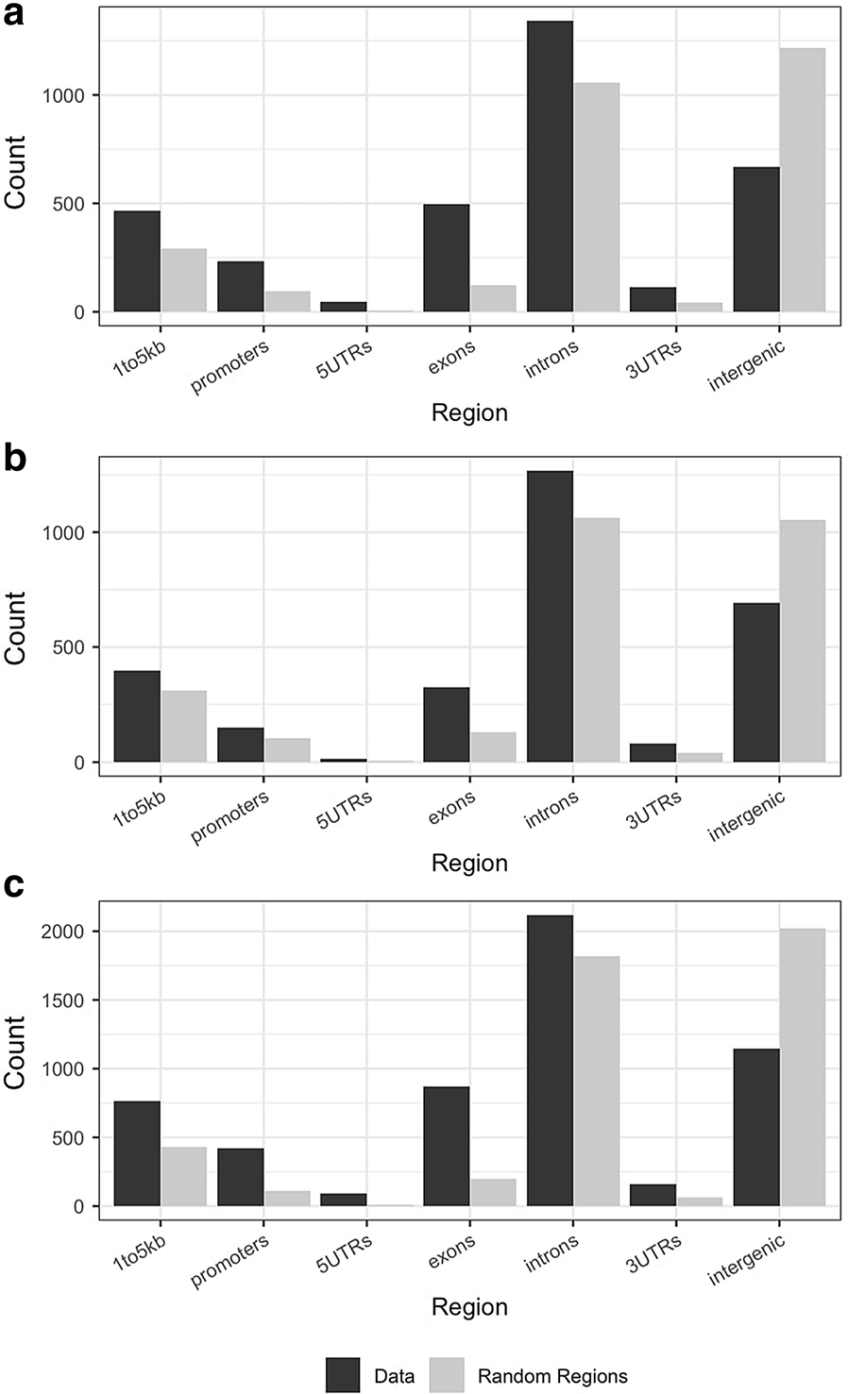
CBD DML distribution in genic regions. Distribution of differentially methylated loci (DML) counts in seven genic regions for **a** directly exposed F0 female cerebral cortex, **b** developmentally exposed F1 female cerebral cortex, and **c** developmentally exposed F1 female hippocampus compared to random distributions generated by the R package annotatr. Experimentally detected loci are shown in black, and randomized loci are in gray

In order to identify genome-wide DNA methylation effects at the single nucleotide level in both directly and developmentally exposed animals, reduced representation bisulfite sequencing (RRBS) was applied to female F0 and F1 wild-type *a/a* cerebral cortex and F1 wild-type hippocampus. Differential methylation comparisons revealed 4190 differentially methylated loci (DMLs) in F1 hippocampus and 2234 DMLs in F1 cerebral cortex. Both F1 tissues exhibited a bias toward hypomethylation overall with 66.6% of DMLs being hypomethylated in hippocampus and 60.6% being hypomethylated in cortex (Fig. 2). In order to determine direct effects of CBD on the epigenome, genome-wide DNA methylation was also assessed in the cerebral cortex of chronically exposed F0 females. 2523 DMLs were identified in F0 cortex with 55% of DMLs being hypomethylated. Randomization of F1 and F0 DMLs using the R package *annotatr* revealed significant enrichment in genic regions including promoters, exons, and 5′ and 3′ untranslated regions for all three tissues (*p* < 0.01; Fig. 3).

To evaluate potential functional molecular consequences of CBD exposure, genes containing the largest number of DMLs in each tissue were identified (Table 1).

**Table 1:** Top DML-containing genes and mean methylation change values

Tissue	Gene	DMLs	Mean methylation (%)	SD
F1 Hippocampus	*Tnxb*	9	28.6	43.6
	*Ncor2*	8	− 35.4	51.1
	*Prdm16*	8	− 5.21	61.1
	*Zfhx3*	8	− 19.2	47.9
	*Gse1*	7	− 59.7	13.9
F1 Cortex	*Tmem151b*	8	55.1	8.5
	*Prdm16*	7	− 65.4	13.2
	*Epas1*	6	68.7	11.7
	*Fhl1*	6	− 51.6	5.9
	*Gse1*	6	− 40.0	54.6
F0 Cortex	*Camta1*	9	− 13.8	59.7
	*Grip1*	7	− 43.2	39.8
	*Cask*	5	− 42.4	54.1
	*Gdf1*	5	− 53.4	6.0
	*Mfsd12*	5	60.2	11.3

Mean methylation values (percent, exposed—control) for F1 and F0 brain regions. The mean methylation column represents the average change across all called DMLs for a given gene. Standard deviation represents the variability between DMLs within a gene

No multi-CpG differentially methylated regions (DMRs) were identified in any tissue, likely due to stringent criteria for DMR calling. Notable genes containing a high number of DMLs in F1 hippocampus included nuclear receptor corepressor 2 (*Ncor2*), which contained eight primarily hypomethylated DMLs in introns and the 1–5 Kb upstream region (Additional file 7). Loss of *Ncor2* function has been associated with memory impairment and reduced social interactions via altered GABAergic signaling in mice [84–86]. The histone methyltransferase PR domain-containing 16 (*Prdm16*) contained seven intronic DMLs and one 32.9% hypomethylated promoter DML. Shimada et al. determined that *Prdm16* knockout is neonatally lethal and the gene is required for neural stem cell maintenance and neurogenesis in the postnatal hippocampal dentate gyrus [87]. In F1 cortex, transmembrane protein 151B (*Tmem151b*) contained the largest number of DMLs. The function of this gene is not well understood; however, one study associated *Tmem151b* knockout with lowered seizure threshold in mice [88]. Eight hypermethylated DMLs, all exonic, were identified in *Tmem151b* in the present study. *Prdm16* also contained seven hypomethylated DMLs in F1 cortex in addition to those found in F1 hippocampus. Additionally, T-lymphoma invasion and metastasis-inducing protein 1 (*Tiam1*), a gene found to be involved in memory storage in a knockout mouse model [89], contained three hypermethylated and three hypomethylated intronic DMLs. Notably, autism susceptibility candidate 2 (*Auts2*) contained five hypomethylated intronic DMLs in F1 cortex; exonic mutations in this gene cause a syndromic form of intellectual disability through its involvement in neuronal migration, neuritogenesis, and transcriptional regulation [90–93]. In F1 hippocampus, DNA methyltransferase 1 (*Dnmt1*) contained one intronic DML that was 57% hypomethylated in exposed animals. DMLs were not identified in *Dnmt1* or any other DNA methyltransferases in F1 cortex or F0 cortex.

In F0 cortex, top DML-containing genes were also identified. Genes containing the largest number of DMLs included calmodulin-binding transcription activator 1 (*Camta1*), which is involved in long-term and episodic memory and has been correlated with intellectual disability when mutated [94–97]. In the present study, *Camta1* contained eight intronic DMLs and one promoter DML, which were largely hypomethylated. Additionally, glutamate receptor-interacting protein 1 (*Grip1*) contained four hypomethylated DMLs in the 1–5 Kb upstream region and three intronic DMLs in directly exposed F0 mice. In previous studies, *Grip1* knockout mice exhibited increased sociability and human *GRIP1* gain of function mutations have been correlated with more severe social deficits in autism spectrum disorder [98, 99]. Interestingly, direct CBD exposure also resulted in both hypermethylation and hypomethylation of CpGs in long interspersed nuclear element (LINE1) retrotransposons in F0 females, while intracisternal A particle (IAP) retrotransposons were unaffected (Additional file 8). Multiple genes known to interact either directly or indirectly with exogenous cannabinoids that are hypothesized to mediate some of CBD’s effects (endocannabinoid, PPARγ, and TRPV receptors) did not contain RRBS DMLs in either generation.

Gene Ontology, phenotype, and disease terms were analyzed for overrepresentation in the list of DML-containing genes for each F1 and F0 tissue. The top significantly enriched terms for each tissue are presented in Table 2, and the full results are available in Additional file 9. Top-enriched Gene Ontology terms for F0 and F1 tissues included terms related to neurogenesis, neuron differentiation and projection, ion transport, and synaptic and postsynaptic cellular components. CBD’s positive effects on neurogenesis are well established [4, 7, 16, 20, 100, 101], and THC has been shown to modify neuron morphology in multiple brain regions including the nucleus accumbens, an area commonly associated with drug-related reward [102]. Prominent overrepresented mouse phenotypes for F0 and F1 DML-containing genes included abnormal synaptic transmission and neuron morphology. Lastly, neurodevelopmental disorders, intellectual disability, epilepsy, and autistic disorders were among the most enriched disease terms. In F1 hippocampus drug abuse terms were the most numerous despite not appearing in the top five terms, with terms related to addiction and substance use occupying eight of the top twenty disease term enrichment positions (Additional file 9).

**Table 2:** ToppFun top 5 enriched functional terms by tissue, significant with Bonferroni-corrected *p* values < 0.05

Tissue	GO: Molecular function	GO: Biological process	GO: Cellular component	Mouse phenotype	Disease
F1 Hippocampus	1. Metal ion transmembrane transporter activity (GO:0046873)	1. Neurogenesis (GO:0022008)	1. Synapse (GO:0045202)	1. Abnormal synaptic transmission (MP:0003635)	1. Neurodevelopmental disorders (C1535926)
	2. Cation channel activity (GO:0005261)	2. Generation of neuron (GO:0048699)	2. Neuron projection (GO:0043005)	2. Abnormal brain morphology (MP:0002152)	2. Intellectual disability (C3714756)
	3. Ion channel activity (GO:0005216)	3. Neuron differentiation (GO:0030182)	3. Cell junction (GO:0030054)	3. Abnormal CNS synaptic transmission (MP:0002206)	3. Epilepsy (C0014544)
	4. Cation transmembrane transporter activity (GO:0008324)	4. Neuron development (GO:0048666)	4. Somatodendritic compartment (GO:0036477)	4. Abnormal neuron morphology (MP:0002882)	4. Autistic disorder (C0004352)
	5. Inorganic cation transmembrane transporter activity (GO:0022890)	5. Cell–cell signaling (GO:0007267)	5. Synaptic membrane (GO:0097060)	5. Abnormal lymphatic vessel endothelium morphology (MP:0010195)	5. Global developmental delay (C0557874)
F1 Cortex	1. Actin binding (GO:0003779)	1. Cell morphogenesis (GO:0000902)	1. Synapse (GO:0045202)	1. Abnormal CNS synaptic transmission (MP:0002206)	1. Intellectual disability (C3714756)
	2. Cytoskeletal protein binding (GO:0008092)	2. Neurogenesis (GO:0022008)	2. Synaptic membrane (GO:0097060)	2. Abnormal synaptic transmission (MP:0003635)	2. Neurodevelopmental disorders (C1535926)
	3. Regulatory region nucleic acid binding (GO:0001067)	3. Cellular component morphogenesis (GO:0032989)	3. Neuron projection (GO:0043005)		3. Global developmental delay (C0557874)
	4. Channel activity (GO:0015267)	4. Neuron differentiation (GO:0030182)	4. Postsynapse (GO:0098794)		4. Developmental delay (disorder) (C0424605)
	5. Passive transmembrane transporter activity (GO:0022803)	5. Generation of neurons (GO:0048699)	5. Postsynaptic density (GO:0014069)		5. Congenital abnormality (C0000768)
F0 Cortex	1. Channel activity (GO:0015267)	1. Neurogenesis (GO:0022008)	1. Neuron projection (GO:0043005)	1. Abnormal CNS synaptic transmission (MP:0002206)	1. Intellectual disability (C3714756)
	2. Passive transmembrane transporter activity (GO:002280)	2. Generation of neurons (GO:0048699)	2. Synapse (GO:0045202)	2. Abnormal synaptic transmission (MP:0003635)	2. Epilepsy (C0014544)
	3. Ion channel activity (GO:0005216)	3. Cellular component morphogenesis (GO:0032989)	3. Postsynapse (GO:0098794)	3. Abnormal locomotor behavior (MP:0001392)	3. Autistic disorder (C0004352)
	4. Ion transmembrane transporter activity (GO:0015075)	4. Cell morphogenesis (GO:0000902)	4. Somatodendritic compartment (GO:0036477)	4. Abnormal brain morphology (MP:0002152)	4. Neurodevelopmental disorders (C1535926)
	5. Cation channel activity (GO:0005261)	5. Neuron differentiation (GO:0030182)	5. Cell junction (GO:0030054)	5. Abnormal locomotor activation (MP:0003313)	5. Bipolar disorder (C0005586)

## Discussion

There is increasing interest in the beneficial effects of cannabinoids for psychological phenotypes as well as for pregnancy-related concerns such as hyperemesis. This combination warrants careful investigation regarding the potential impacts of exposure to CBD during development, especially given the known negative effects of prenatal THC. The main finding of the present study is that developmental CBD exposure in mice is associated with widespread changes in the brain methylome and sex-specific effects on anxiety and memory behavior. Direct exposure during pregnancy and lactation also modifies DNA methylation with a similar magnitude but does not impart changes in memory or anxiety. Together, these data suggest that despite its previously studied benefits in direct exposure CBD use during pregnancy may have negative consequences for adult offspring, though the observed effects were mixed. Additionally, based on functional enrichment of DML-containing genes it is possible that neuroepigenetic mechanisms are mechanistically involved in developmental and direct CBD exposure’s behavioral effects; additional studies will be required to more directly link these phenomena.

The present study represents the first interrogation of developmental CBD’s impact on offspring psychological phenotypes. Developmental exposure to CBD as seen in F1 behavior results in sex-specific increases in anxiety and memory performance. These results parallel some findings for prenatal exposure to the psychoactive cannabinoid THC and contrast others. Trezza et al. found that prenatal THC resulted in an anxiogenic profile in adult offspring as measured by the elevated plus maze; however, Manduca et al. did not find anxiety or other behavioral abnormalities in adulthood despite neonatal differences in vocalization behavior with prenatal exposure to the synthetic cannabinoid WIN55212-2 [24, 103]. Silva et al. and de Salas-Quiroga et al. identified memory deficits with prenatal THC exposure [22, 104], which contrast with improved memory function observed in the present study; however, small sample size and the removal of outliers are important considerations and warrant further investigation into the effect. Other behavioral phenotypes for prenatal THC exposure have also been observed in animals, including altered cognitive function, emotional reactivity, and responses to drugs of abuse such as methamphetamine and opioids [24, 26, 28, 104], warranting expansion of prenatal CBD studies to these areas. Importantly, the marble-burying assay employed in the current study has significant limitations, specifically that it remains unclear whether the task measures novelty-induced anxiety or compulsive/repetitive behavior [72, 105]. Subsequent studies of prenatal CBD exposure should employ additional measures of anxiety such as the elevated plus maze or open-field tests as well as measures of compulsivity such as nestlet shredded to provide greater phenotypic accuracy. The observed sex-specific nature of developmental CBD’s behavioral effects remains notable; however, investigation of differential methylation in both males and females following prenatal exposure (as opposed to assaying females only as in the current study) may help identify vulnerabilities or protective effects in each sex. Interestingly, effects on wean weight or postweaning weight for CBD were not observed in the present study in contrast to THC, which has been shown to reduce wean weight in rats [106] and reduce birth weight in rats [107] and humans [108] despite evidence for hyperphagia with direct exposure [109–111]. A lack of significant behavioral effects in the directly exposed F0 generation corresponds with previous studies such as Fogaça et al. and Gáll et al. which identified anxiolytic effects for CBD in the presence of chronic unpredictable stress but not in its absence [101, 112]. In contrast to these studies, the extended exposure window from prior to breeding through lactation employed here prevents identification of the discrete window of exposure mediating the observed changes and will require additional study. Regarding routes of prenatal exposure, cannabinoids are known to rapidly cross the placenta and are found in breast milk due to their high lipophilicity [113–117]; a study by Feinshtein et al. additionally found that CBD increased the permeability of the placenta to other xenobiotics [118].

Genome-wide DNA methylation results identified thousands of DMLs enriched in genic regions in each generation and brain region. These findings expand upon findings by Pucci et al. and Paradisi et al. who initially identified DNA methylation changes with exposure to CBD and the endocannabinoid anandamide, respectively, in keratinocytes in vitro [119, 120]. These studies found hypermethylation associated with CBD exposure in contrast to the current study, where hypomethylation was more predominant in both directly and developmentally exposed animals. Inclusion of only females in the F1 RRBS analysis prevents sex comparisons on the epigenetic level, and future studies will be needed to determine whether behavior differences are reflected in differential methylation between males and females. A recent study by Sales et al., who identified changes in global DNA methylation levels in the mouse prefrontal cortex and hippocampus following direct CBD exposure, also associated CBD with hypermethylation by finding that the compound restored hypomethylation triggered by unpredictable stress [2]. While a mixture of hyper- and hypomethylation was identified in the present study, the slightly greater prominence of hypomethylation (particularly in the F1 hippocampus) agrees with our previous study of brain methylation with direct CBD exposure in male mice [75]. While site-specific methylation changes may have positive or negative effects on gene expression depending on genic region, binding of methylation readers and other factors, hypomethylation is generally associated with increased chromosomal instability, activation of transposons, and reduced cell survival in CNS neurons [121–124]. Further study is needed to determine whether transposable element methylation is reduced by prenatal CBD; however, our finding that direct CBD results in mixed differential methylation within LINE1 retrotransposons indicates that transposon methylation patterns may not correspond with genic trends. One limitation of the current study that is highly relevant to both DNA methylation and behavior outcomes is that the effects of CBD on maternal care are unknown. Differences in maternal care have been shown to impact both offspring behavior and brain DNA methylation in the hippocampus and other brain regions [125–131]. Continuous infusion of the THC-like cannabinoid receptor agonist WIN-55212-2 has been associated with reduced maternal care during lactation [132]; further study of CBD in the context of maternal care will be required to delineate its effects from those of developmental exposure.

Functional enrichment of F1 DML-containing genes revealed overrepresentation of neurogenesis, neuron morphology, and metal ion channel terms, while top disease terms included autism spectrum disorder, schizophrenia, and intellectual disability. Direct exposure to CBD has been shown to improve anxiety and memory behavior in rodents [12, 133–137] and reduce psychotic symptoms in rodent models and humans [9, 10, 138, 139], and it appears that similar pathways are affected on the epigenetic level with developmental exposure. Based on behavior results, it can be hypothesized that prenatal exposure affects these pathways in a disruptive manner; however, further characterization of prenatal CBD’s behavioral effects will be required to validate the observed results. Neurogenesis was a particularly enriched term in hippocampus, and while it is not possible from these data to determine which window of neurogenesis (embryogenic, postnatal, or adult) was affected by exposure, it is important to note that CBD has been shown to stimulate adult neurogenesis in the hippocampal dentate gyrus [7, 16, 20, 100, 140]. Whether altered DNA methylation in neurogenesis pathways in the adult F1 hippocampus represents changes to ongoing adult neurogenesis or signatures of perturbation earlier in development will be a highly relevant distinction based on the differential consequences of increased neurogenesis during these windows. Enhanced adult neurogenesis has been associated with exercise, environmental enrichment, and reduced depressive symptoms [141–145], while regional increases during the developmental and postnatal windows are linked to diseases such as autism spectrum disorder [146–148].

## Conclusions

Overall, the current study identified sex-specific changes in working spatial memory and anxiety behavior as well as genome-wide changes in brain DNA methylation in adult mouse offspring developmentally exposed to human-relevant doses of CBD. The collected data represent an initial inquiry into the effects of prenatal CBD exposure on the adult brain and behavioral phenotypes, indicating that significant efforts are needed to fully characterize the impacts of this compound during development.

## Supplementary Information

**Additional file 1:** Primer information for pyrosequencing of LINE1 and IAP transposons.

**Additional file 2:** Body weight trends for wild-type a/a F1 offspring of both sexes from weaning through study completion (12 weeks). Shaded areas represent 95% confidence intervals. Weights did not differ significantly between groups at any time point by ANOVA (p = 0.995).

**Additional file 3:** Litter size for F1 pups differed significantly between groups with CBD-exposed litters containing 1.25 more pups on average in comparison to control litters (p = 0.0134).

**Additional file 4:** Scores for wild-type a/a F1 young adult mice in (a) marble burying, a measure of anxiety and (b) Y-maze spontaneous alternation and (c) Y-maze arm entries, measures of spatial memory, did not differ significantly between CBD-exposed and control groups when results from both sexes were combined.

**Additional file 5:** Scores for wild-type a/a F0 adult female mice in (a) marble burying, a measure of anxiety and (b) Y-maze spontaneous alternation and (c) Y-maze arm entries, measures of spatial memory and locomotion, did not differ significantly between animals receiving 20 mg/kg CBD daily for 9 weeks and controls for either acute (test performed near CBD Cmax) or cumulative (test performed 24 hours after last dose) runs. Likewise, paired comparisons for acute vs. cumulative scores were not statistically significant.

**Additional file 6:** Measurements of F1 Avy/a offspring coat color on a three category scale (L: low methylation/yellow; M: medium methylation/mottled; H: high methylation/pseudoagouti) revealed a lack of statistically significant differences between CBD-exposed and control groups when assessed (a) as a whole and (b) stratified by sex.

**Additional file 7:** Table of all unique, statistically significant differentially methylated loci (DMLs) identified between CBD-exposed and control tissues consisting of F0 cerebral cortex, F1 cerebral cortex, and F1 hippocampus.

**Additional file 8:** Methylation values (four CpG positions and mean) for CpGs lying in (a) mLINE1 and (b) IAP retrotransposons in F0 female cerebral cortex and hippocampus. Significant hypermethylation was identified at mLINE1 position 1 and hypomethylation at mLINE1 position 2 in CBD-exposed tissues, both in cerebral cortex only. Other mLINE1 positions, mean methylation, and IAP positions and mean were not found to be significantly different between groups. Each point represents an individual animal. * = p < 0.05, ** = p < 0.01.

**Additional file 9:** Complete results returned by the functional enrichment tool ToppFun for lists of DML-containing genes in F0 cerebral cortex, F1 cerebral cortex, and F1 hippocampus.

## Data Availability

The raw datasets generated during the current study are available in the Sequence Read Archive repository at Accession No. PRJNA655745; https://www.ncbi.nlm.nih.gov/sra/PRJNA655745. Processed datasets generated during the current study are available as Additional files.

## Abbreviations

CBD: Cannabidiol
THC: Δ9-Tetrahydrocannabinol
5HT-1a: 5-Hydroxytyptamine (serotonin) 1a
PPARγ: Peroxisome proliferator-activated receptor gamma
TRPV: Transient receptor potential cation channel subfamily V
GABA: Gamma aminobutyric acid
DOHaD: Developmental Origins of Health and Disease
*A*^*vy*^: Agouti viable yellow
RRBS: Reduced-representation bisulfite sequencing
IAP: Intracisternal A particle
ANOVA: Analysis of variance
gDNA: Genomic deoxyribonucleic acid
LINE1: Murine long interspersed nuclear element 1
DML: Differentially methylated locus
DMR: Differentially methylated region
F0: Filial generation 0
F1: Filial generation 1
Ncor2: Nuclear receptor corepressor 2
Prdm16: PR domain-containing 16
Tmem151b: Transmembrane protein 151B
Tiam1: T-lymphoma invasion and metastasis-inducing protein 1
Auts2: Autism susceptibility candidate 2
Dnmt1: DNA methyltransferase 1
Camta1: Calmodulin-binding transcription activator 1
Grip1: Glutamate receptor-interacting protein 1

## References

[1] Total CBD consumer sales US 2014–2022 | Statista [Internet] Statista. [cited 2020 Mar 18]. Available from: https://www.statista.com/statistics/760498/total-us-cbd-sales/.

[2] Sales AJ, Guimarães FS, Joca SRL (2020). CBD modulates DNA methylation in mice prefrontal cortex and hippocampus of mice exposed to forced swim. Behav Brain Res [Internet].

[3] Réus GZ, Stringari RB, Ribeiro KF, Luft T, Abelaira HM, Fries GR (2011). Administration of cannabidiol and imipramine induces antidepressant-like effects in the forced swimming test and increases brain-derived neurotrophic factor levels in the rat amygdala. Acta Neuropsychiatr [Internet].

[4] Luján MÁ, Castro-Zavala A, Alegre-Zurano L, Valverde O (2018). Repeated Cannabidiol treatment reduces cocaine intake and modulates neural proliferation and CB1R expression in the mouse hippocampus. Neuropharmacology [Internet].

[5] Campos AC, Guimarães FS (2008). Involvement of 5HT1A receptors in the anxiolytic-like effects of cannabidiol injected into the dorsolateral periaqueductal gray of rats. Psychopharmacology [Internet].

[6] Resstel LBM, Tavares RF, Lisboa SFS, Joca SRL, Corrêa FMA, Guimarães FS (2009). 5-HT1A receptors are involved in the cannabidiol-induced attenuation of behavioural and cardiovascular responses to acute restraint stress in rats. Br J Pharmacol [Internet].

[7] Schiavon AP, Bonato JM, Milani H, Guimarães FS, Weffort de Oliveira RM (2016). Influence of single and repeated cannabidiol administration on emotional behavior and markers of cell proliferation and neurogenesis in non-stressed mice. Prog Neuropsychopharmacol Biol Psychiatry [Internet].

[8] Katsidoni V, Anagnostou I, Panagis G (2013). Cannabidiol inhibits the reward-facilitating effect of morphine: involvement of 5-HT1A receptors in the dorsal raphe nucleus. Addict Biol [Internet].

[9] Leweke FM, Piomelli D, Pahlisch F, Muhl D, Gerth CW, Hoyer C (2012). Cannabidiol enhances anandamide signaling and alleviates psychotic symptoms of schizophrenia. Transl Psychiatry [Internet].

[10] McGuire P, Robson P, Cubala WJ, Vasile D, Morrison PD, Barron R (2018). Cannabidiol (CBD) as an adjunctive therapy in schizophrenia: a multicenter randomized controlled trial. Am J Psychiatry [Internet].

[11] Bergamaschi MM, Queiroz RHC, Chagas MHN, de Oliveira DCG, De Martinis BS, Kapczinski F (2011). Cannabidiol reduces the anxiety induced by simulated public speaking in treatment-naïve social phobia patients. Neuropsychopharmacology [Internet].

[12] Zuardi AW, Cosme RA, Graeff FG, Guimarães FS (1993). Effects of ipsapirone and cannabidiol on human experimental anxiety. J Psychopharmacol [Internet].

[13] Zuardi AW, Shirakawa I, Finkelfarb E, Karniol IG (1982). Action of cannabidiol on the anxiety and other effects produced by delta 9-THC in normal subjects. Psychopharmacology [Internet].

[14] Di Forti M, Morgan C, Dazzan P, Pariante C, Mondelli V, Marques TR (2009). High-potency cannabis and the risk of psychosis. Br J Psychiatry [Internet].

[15] Niesink RJM, van Laar MW (2013). Does cannabidiol protect against adverse psychological effects of THC?. Front Psychiatry [Internet].

[16] Campos AC, Ortega Z, Palazuelos J, Fogaça MV, Aguiar DC, Díaz-Alonso J (2013). The anxiolytic effect of cannabidiol on chronically stressed mice depends on hippocampal neurogenesis: involvement of the endocannabinoid system. Int J Neuropsychopharmacol [Internet].

[17] Vallée A, Lecarpentier Y, Guillevin R, Vallée J-N (2017). Effects of cannabidiol interactions with Wnt/β-catenin pathway and PPARγ on oxidative stress and neuroinflammation in Alzheimer’s disease. Acta Biochim Biophys Sin [Internet].

[18] Pumroy RA, Samanta A, Liu Y, Hughes TE, Zhao S, Yudin Y (2019). Molecular mechanism of TRPV2 channel modulation by cannabidiol. Elife [Internet].

[19] Hampson AJ, Grimaldi M, Axelrod J, Wink D. Cannabidiol and (-)Δ9-tetrahydrocannabinol are neuroprotective antioxidants. Proc Natl Acad Sci U S A [Internet] 1998;95(14):8268–73. Available from: https://www.scopus.com/inward/record.uri?eid=2-s2.0-0032493197&doi=10.1073%2fpnas.95.14.8268&partnerID=40&md5=386cd2de325f0e8404375f38807a18d6. 10.1073/pnas.95.14.8268 PMC20965 9653176

[20] Luján MÁ, Cantacorps L, Valverde O (2019). The pharmacological reduction of hippocampal neurogenesis attenuates the protective effects of cannabidiol on cocaine voluntary intake. Addict Biol [Internet].

[21] Wanner NM, Colwell ML, Faulk C. The epigenetic legacy of illicit drugs: developmental exposures and late-life phenotypes. Environ Epigenet [Internet] 2019 Oct 1 [cited 2019 Nov 26];5(4). Available from: https://academic.oup.com/eep/article-pdf/5/4/dvz022/31109423/dvz022.pdf. 10.1093/eep/dvz022 PMC6875650 31777665

[22] de Salas-Quiroga A, García-Rincón D, Gómez-Domínguez D, Valero M, Simón-Sánchez S, Paraíso-Luna J, et al. Long-term hippocampal interneuronopathy drives sex-dimorphic spatial memory impairment induced by prenatal THC exposure. Neuropsychopharmacology [Internet] 2020; 10.1038/s41386-020-0621-3. 10.1038/s41386-020-0621-3 PMC7075920 31982904

[23] de Salas-Quiroga A, Díaz-Alonso J, García-Rincón D, Remmers F, Vega D, Gómez-Cañas M (2015). Prenatal exposure to cannabinoids evokes long-lasting functional alterations by targeting CB_1_ receptors on developing cortical neurons. Proc Natl Acad Sci USA [Internet].

[24] Trezza V, Campolongo P, Cassano T, Macheda T, Dipasquale P, Carratù MR (2008). Effects of perinatal exposure to delta-9-tetrahydrocannabinol on the emotional reactivity of the offspring: a longitudinal behavioral study in Wistar rats. Psychopharmacology [Internet].

[25] Mereu G, Fà M, Ferraro L, Cagiano R, Antonelli T, Tattoli M (2003). Prenatal exposure to a cannabinoid agonist produces memory deficits linked to dysfunction in hippocampal long-term potentiation and glutamate release. Proc Natl Acad Sci USA [Internet].

[26] Spano MS, Ellgren M, Wang X, Hurd YL (2007). Prenatal cannabis exposure increases heroin seeking with allostatic changes in limbic enkephalin systems in adulthood. Biol Psychiatry [Internet].

[27] Vela G, Martín S, García-Gil L, Crespo JA, Ruiz-Gayo M, Fernández-Ruiz JJ (1998). Maternal exposure to delta9-tetrahydrocannabinol facilitates morphine self-administration behavior and changes regional binding to central mu opioid receptors in adult offspring female rats. Brain Res [Internet].

[28] DiNieri JA, Wang X, Szutorisz H, Spano SM, Kaur J, Casaccia P (2011). Maternal cannabis use alters ventral striatal dopamine D2 gene regulation in the offspring. Biol Psychiatry [Internet].

[29] Pérez-Rosado A, Manzanares J, Fernández-Ruiz J, Ramos JA (2000). Prenatal Delta(9)-tetrahydrocannabinol exposure modifies proenkephalin gene expression in the fetal rat brain: sex-dependent differences. Brain Res Dev Brain Res [Internet].

[30] Castaldo P, Magi S, Cataldi M, Arcangeli S, Lariccia V, Nasti AA (2010). Altered regulation of glutamate release and decreased functional activity and expression of GLT1 and GLAST glutamate transporters in the hippocampus of adolescent rats perinatally exposed to Delta(9)-THC. Pharmacol Res [Internet].

[31] Beggiato S, Borelli AC, Tomasini MC, Morgano L, Antonelli T, Tanganelli S (2017). Long-lasting alterations of hippocampal GABAergic neurotransmission in adult rats following perinatal Δ9-THC exposure. Neurobiol Learn Mem [Internet].

[32] Molina-Holgado F, Amaro A, González MI, Alvarez FJ, Leret ML (1996). Effect of maternal delta 9-tetrahydrocannabinol on developing serotonergic system. Eur J Pharmacol [Internet].

[33] Wadhwa PD, Buss C, Entringer S, Swanson JM (2009). Developmental origins of health and disease: brief history of the approach and current focus on epigenetic mechanisms. Semin Reprod Med [Internet].

[34] Barker DJP (2007). The origins of the developmental origins theory. J Intern Med [Internet].

[35] Barker DJ. Fetal origins of coronary heart disease. BMJ [Internet] 1995 Jul 15;311(6998):171–4. Available from: https://www.ncbi.nlm.nih.gov/pubmed/7613432. 10.1136/bmj.311.6998.171 PMC2550226 7613432

[36] Barker DJ, Osmond C (1986). Infant mortality, childhood nutrition, and ischaemic heart disease in England and Wales. Lancet [Internet].

[37] Wockner LF, Noble EP, Lawford BR, Young RM, Morris CP, Whitehall VLJ (2014). Genome-wide DNA methylation analysis of human brain tissue from schizophrenia patients. Transl Psychiatry [Internet].

[38] Chen J, Zang Z, Braun U, Schwarz K, Harneit A, Kremer T (2020). Association of a reproducible epigenetic risk profile for schizophrenia with brain methylation and function. JAMA Psychiatry [Internet].

[39] Zhao H, Xu J, Pang L, Zhang Y, Fan H, Liu L (2015). Genome-wide DNA methylome reveals the dysfunction of intronic microRNAs in major psychosis. BMC Med Genomics [Internet].

[40] Garcia-Ruiz B, Moreno L, Muntané G, Sánchez-Gistau V, Gutiérrez-Zotes A, Martorell L (2020). Leukocyte and brain DDR1 hypermethylation is altered in psychosis and is correlated with stress and inflammatory markers. Epigenomics [Internet].

[41] Dong E, Ruzicka WB, Grayson DR, Guidotti A (2015). DNA-methyltransferase1 (DNMT1) binding to CpG rich GABAergic and BDNF promoters is increased in the brain of schizophrenia and bipolar disorder patients. Schizophr Res [Internet].

[42] Smigielski L, Jagannath V, Rössler W, Walitza S, Grünblatt E (2020). Epigenetic mechanisms in schizophrenia and other psychotic disorders: a systematic review of empirical human findings. Mol Psychiatry [Internet].

[43] Zheng Y, Fan W, Zhang X, Dong E (2016). Gestational stress induces depressive-like and anxiety-like phenotypes through epigenetic regulation of BDNF expression in offspring hippocampus. Epigenetics [Internet].

[44] Chagnon YC, Potvin O, Hudon C, Préville M (2015). DNA methylation and single nucleotide variants in the brain-derived neurotrophic factor (BDNF) and oxytocin receptor (OXTR) genes are associated with anxiety/depression in older women. Front Genet [Internet].

[45] Swartz JR, Hariri AR, Williamson DE (2017). An epigenetic mechanism links socioeconomic status to changes in depression-related brain function in high-risk adolescents. Mol Psychiatry [Internet].

[46] Payne JL, Osborne LM, Cox O, Kelly J, Meilman S, Jones I, et al. DNA methylation biomarkers prospectively predict both antenatal and postpartum depression. Psychiatry Res [Internet] 2019 Nov 27;112711. Available from: http://www.sciencedirect.com/science/article/pii/S0165178119319535. 10.1016/j.psychres.2019.112711 PMC7702696 31843207

[47] McCoy CR, Glover ME, Flynn LT, Simmons RK, Cohen JL, Ptacek T, et al. Altered DNA methylation in the developing brains of rats genetically prone to high versus low anxiety. J Neurosci [Internet] 2019;39(16):3144–58. Available from: https://www.jneurosci.org/content/39/16/3144.abstract. 10.1523/JNEUROSCI.1157-15.2019 PMC6468100 30683683

[48] Simmons RK, Howard JL, Simpson DN, Akil H, Clinton SM (2012). DNA methylation in the developing hippocampus and amygdala of anxiety-prone versus risk-taking rats. Dev Neurosci [Internet].

[49] McCoy CR, Jackson NL, Day J, Clinton SM (2017). Genetic predisposition to high anxiety- and depression-like behavior coincides with diminished DNA methylation in the adult rat amygdala. Behav Brain Res [Internet].

[50] Schiele MA, Gottschalk MG, Domschke K (2020). The applied implications of epigenetics in anxiety, affective and stress-related disorders—a review and synthesis on psychosocial stress, psychotherapy and prevention. Clin Psychol Rev [Internet].

[51] Nardone S, Sams DS, Reuveni E, Getselter D, Oron O, Karpuj M (2014). DNA methylation analysis of the autistic brain reveals multiple dysregulated biological pathways. Transl Psychiatry [Internet].

[52] Nardone S, Sams DS, Zito A, Reuveni E, Elliott E (2017). Dysregulation of cortical neuron DNA methylation profile in autism spectrum disorder. Cereb Cortex [Internet].

[53] Ladd-Acosta C, Hansen KD, Briem E, Fallin MD, Kaufmann WE, Feinberg AP (2014). Common DNA methylation alterations in multiple brain regions in autism. Mol Psychiatry [Internet].

[54] Corley M, Vargas-Maya N, Pang A, Lum-Jones A, Li D, Khadka V (2019). Epigenetic delay in the neurodevelopmental trajectory of DNA methylation states in autism spectrum disorders. Front Genet [Internet].

[55] Tremblay MW, Jiang Y-H (2019). DNA methylation and susceptibility to autism spectrum disorder. Annu Rev Med [Internet].

[56] Watson CT, Szutorisz H, Garg P, Martin Q, Landry JA, Sharp AJ (2015). Genome-wide DNA methylation profiling reveals epigenetic changes in the rat nucleus accumbens associated with cross-generational effects of adolescent THC exposure. Neuropsychopharmacology [Internet].

[57] Murphy SK, Itchon-Ramos N, Visco Z, Huang Z, Grenier C, Schrott R (2018). Cannabinoid exposure and altered DNA methylation in rat and human sperm. Epigenetics [Internet].

[58] Schrott R, Acharya K, Itchon-Ramos N, Hawkey AB, Pippen E, Mitchell JT (2019). Cannabis use is associated with potentially heritable widespread changes in autism candidate gene DLGAP2 DNA methylation in sperm. Epigenetics [Internet].

[59] Dolinoy DC (2008). The agouti mouse model: an epigenetic biosensor for nutritional and environmental alterations on the fetal epigenome. Nutr Rev [Internet].

[60] Anderson OS, Nahar MS, Faulk C, Jones TR, Liao C, Kannan K (2012). Epigenetic responses following maternal dietary exposure to physiologically relevant levels of bisphenol A. Environ Mol Mutagen [Internet].

[61] Faulk C, Barks A, Liu K, Goodrich JM, Dolinoy DC (2013). Early-life lead exposure results in dose- and sex-specific effects on weight and epigenetic gene regulation in weanling mice. Epigenomics [Internet].

[62] Waterland RA, Jirtle RL (2003). Transposable elements: targets for early nutritional effects on epigenetic gene regulation. Mol Cell Biol [Internet].

[63] Weinhouse C, Anderson OS, Bergin IL, Vandenbergh DJ, Gyekis JP, Dingman MA (2014). Dose-dependent incidence of hepatic tumors in adult mice following perinatal exposure to bisphenol A. Environ Health Perspect [Internet].

[64] Küster T, Zumkehr B, Hermann C, Theurillat R, Thormann W, Gottstein B, et al. Voluntary ingestion of antiparasitic drugs emulsified in honey represents an alternative to gavage in mice. J Am Assoc Lab Anim Sci [Internet] 2012; 51(2):219–23. Available from: https://www.ncbi.nlm.nih.gov/pubmed/22776122 PMC3314525 22776122

[65] Nair AB, Jacob S (2016). A simple practice guide for dose conversion between animals and human. J Basic Clin Physiol Pharmacol [Internet].

[66] El-Alfy AT, Ivey K, Robinson K, Ahmed S, Radwan M, Slade D, et al. Antidepressant-like effect of Δ9-tetrahydrocannabinol and other cannabinoids isolated from Cannabis sativa L. Pharmacol Biochem Behav [Internet] 2010;95(4):434–42. Available from: http://www.sciencedirect.com/science/article/pii/S0091305710000730. 10.1016/j.pbb.2010.03.004 PMC2866040 20332000

[67] National Research Council (US) Committee for the Update of the Guide for the Care and Use of Laboratory Animals. Guide for the Care and Use of Laboratory Animals [Internet] Washington (DC): National Academies Press (US); 2011. 10.17226/12910 21595115

[68] Deiana S, Watanabe A, Yamasaki Y, Amada N, Arthur M, Fleming S (2012). Plasma and brain pharmacokinetic profile of cannabidiol (CBD), cannabidivarine (CBDV), Δ^9^-tetrahydrocannabivarin (THCV) and cannabigerol (CBG) in rats and mice following oral and intraperitoneal administration and CBD action on obsessive-compulsive behaviour. Psychopharmacology [Internet].

[69] Tolman EC. Purpose and cognition: the determiners of animal learning. Psychol Rev [Internet] 1925; 32(4):285–97. Available from: https://psycnet.apa.org/fulltext/1927-00608-001.pdf

[70] Hughes RN (2004). The value of spontaneous alternation behavior (SAB) as a test of retention in pharmacological investigations of memory. Neurosci Biobehav Rev [Internet].

[71] Murphy M, Mills S, Winstone J, Leishman E, Wager-Miller J, Bradshaw H, et al. Chronic adolescent Δ9-tetrahydrocannabinol treatment of male mice leads to long-term cognitive and behavioral dysfunction, which are prevented by concurrent cannabidiol treatment. Cannabis Cannabinoid Res [Internet] 2017;2(1):235–46. Available from: https://doi.org/10.1089/can.2017.0034?casa_token=qMf-DUawBoUAAAAA:_BOei0nEynrQ6Ut-U1Ganhna6AMe-2fFQILu0wYZ0yTa54Y8slksWPMuJvAprIiDZm5Gd3PZLPFA0Q . 10.1089/can.2017.0034 PMC5655843 29098186

[72] Angoa-Pérez M, Kane MJ, Briggs DI, Francescutti DM, Kuhn DM (2013). Marble burying and nestlet shredding as tests of repetitive, compulsive-like behaviors in mice. J Vis Exp [Internet].

[73] Deacon RMJ (2006). Digging and marble burying in mice: simple methods for in vivo identification of biological impacts. Nat Protoc [Internet].

[74] Morgan HD, Sutherland HG, Martin DI, Whitelaw E (1999). Epigenetic inheritance at the agouti locus in the mouse. Nat Genet [Internet].

[75] Wanner NM, Colwell M, Drown C, Faulk C (2020). Subacute cannabidiol alters genome-wide DNA methylation in adult mouse hippocampus. Environ Mol Mutagen [Internet].

[76] Spijker S. Dissection of Rodent Brain Regions. In: Li KW, editor. Neuroproteomics [Internet] Totowa, NJ: Humana Press; 2011. p. 13–26. Available from: 10.1007/978-1-61779-111-6_2.

[77] Andrews S (2010). FastQC: a quality control tool for high throughput sequence data.

[78] Krueger F. Trim galore. A wrapper tool around Cutadapt and FastQC to consistently apply quality and adapter trimming to FastQ files. 2015;516:517.

[79] Krueger F, Andrews SR (2011). Bismark: a flexible aligner and methylation caller for Bisulfite–Seq applications. Bioinformatics [Internet].

[80] Park Y, Wu H (2016). Differential methylation analysis for BS-seq data under general experimental design. Bioinformatics [Internet].

[81] Cavalcante RG, Sartor MA. annotatr: Associating genomic regions with genomic annotations [Internet] bioRxiv. 2016 [cited 2020 Jan 29]. p. 039685. Available from: 10.1101/039685v1.abstract.

[82] Chen J, Bardes EE, Aronow BJ, Jegga AG. ToppGene Suite for gene list enrichment analysis and candidate gene prioritization. Nucleic Acids Res [Internet] 2009; 37(Web Server issue):W305–11. 10.1093/nar/gkp427. 10.1093/nar/gkp427 PMC2703978 19465376

[83] Colwell M, Drown M, Showel K, Drown C, Palowski A, Faulk C (2018). Evolutionary conservation of DNA methylation in CpG sites within ultraconserved noncoding elements. Epigenetics [Internet].

[84] Zhou W, He Y, Rehman AU, Kong Y, Hong S, Ding G (2019). Loss of function of NCOR1 and NCOR2 impairs memory through a novel GABAergic hypothalamus-CA3 projection. Nat Neurosci [Internet].

[85] Lyst MJ, Ekiert R, Guy J, Selfridge J, Koerner MV, Merusi C (2018). Affinity for DNA contributes to NLS independent nuclear localization of MeCP2. Cell Rep [Internet].

[86] Lyst MJ, Ekiert R, Ebert DH, Merusi C, Nowak J, Selfridge J (2013). Rett syndrome mutations abolish the interaction of MeCP2 with the NCoR/SMRT co-repressor. Nat Neurosci [Internet].

[87] Shimada IS, Acar M, Burgess RJ, Zhao Z, Morrison SJ (2017). Prdm16 is required for the maintenance of neural stem cells in the postnatal forebrain and their differentiation into ependymal cells. Genes Dev [Internet].

[88] McGarr TC. Gene Identification and Characterization of NMF389, a New Seizure Threshold Mutant [Internet] [M.S.]. Frankel WN, editor. The University of Maine; 2014 [cited 2020 Jul 28]. Available from: https://digitalcommons.library.umaine.edu/etd/2232/.

[89] Kojima H, Rosendale M, Sugiyama Y, Hayashi M, Horiguchi Y, Yoshihara T, et al. The role of CaMKII-Tiam1 complex on learning and memory. Neurobiol Learn Mem [Internet] 2019; 166:107070. 10.1016/j.nlm.2019.107070. 10.1016/j.nlm.2019.107070 31445077

[90] Hori K, Hoshino M. Neuronal Migration and AUTS2 Syndrome. Brain Sci [Internet] 2017 May 14;7(5). 10.3390/brainsci7050054. 10.3390/brainsci7050054 PMC5447936 28505103

[91] Gao Z, Lee P, Stafford JM, von Schimmelmann M, Schaefer A, Reinberg D (2014). An AUTS2-Polycomb complex activates gene expression in the CNS. Nature [Internet].

[92] Beunders G, Voorhoeve E, Golzio C, Pardo LM, Rosenfeld JA, Talkowski ME (2013). Exonic deletions in AUTS2 cause a syndromic form of intellectual disability and suggest a critical role for the C terminus. Am J Hum Genet [Internet].

[93] Hori K, Nagai T, Shan W, Sakamoto A, Taya S, Hashimoto R (2014). Cytoskeletal regulation by AUTS2 in neuronal migration and neuritogenesis. Cell Rep [Internet].

[94] Shinawi M, Coorg R, Shimony JS, Grange DK, Al-Kateb H (2015). Intragenic CAMTA1 deletions are associated with a spectrum of neurobehavioral phenotypes. Clin Genet [Internet].

[95] Bas-Orth C, Tan Y-W, Oliveira AMM, Bengtson CP, Bading H (2016). The calmodulin-binding transcription activator CAMTA1 is required for long-term memory formation in mice. Learn Mem [Internet].

[96] Huentelman MJ, Papassotiropoulos A, Craig DW, Hoerndli FJ, Pearson JV, Huynh K-D (2007). Calmodulin-binding transcription activator 1 (CAMTA1) alleles predispose human episodic memory performance. Hum Mol Genet [Internet].

[97] Magnin E, Blagosklonov O, Sylvestre G, Minot D, Thevenon J, Faivre L (2014). Neuropsychological and neuroimaging phenotype induced by a CAMTA1 mutation. Brain Dev [Internet].

[98] Han M, Mejias R, Chiu S-L, Rose R, Adamczyk A, Huganir R (2017). Mice lacking GRIP1/2 show increased social interactions and enhanced phosphorylation at GluA2-S880. Behav Brain Res [Internet].

[99] Mejias R, Adamczyk A, Anggono V, Niranjan T, Thomas GM, Sharma K (2011). Gain-of-function glutamate receptor interacting protein 1 variants alter GluA2 recycling and surface distribution in patients with autism. Proc Natl Acad Sci USA [Internet].

[100] Esposito G, Scuderi C, Valenza M, Togna GI, Latina V, De Filippis D (2011). Cannabidiol reduces Aβ-induced neuroinflammation and promotes hippocampal neurogenesis through PPARγ involvement. PLoS ONE [Internet].

[101] Fogaça MV, Campos AC, Coelho LD, Duman RS, Guimarães FS (2018). The anxiolytic effects of cannabidiol in chronically stressed mice are mediated by the endocannabinoid system: role of neurogenesis and dendritic remodeling. Neuropharmacology [Internet].

[102] Kolb B, Li Y, Robinson T, Parker LA (2018). THC alters alters morphology of neurons in medial prefrontal cortex, orbital prefrontal cortex, and nucleus accumbens and alters the ability of later experience to promote structural plasticity. Synapse [Internet].

[103] Manduca A, Servadio M, Melancia F, Schiavi S, Manzoni OJ, Trezza V. Sex-specific behavioural deficits induced at early life by prenatal exposure to the cannabinoid receptor agonist WIN55, 212-2 depend on mGlu5 receptor signalling. Br J Pharmacol [Internet]. 2020; 177(2):449–63. 10.1111/bph.14879. 10.1111/bph.14879 PMC6989958 31658362

[104] Silva L, Zhao N, Popp S, Dow-Edwards D (2012). Prenatal tetrahydrocannabinol (THC) alters cognitive function and amphetamine response from weaning to adulthood in the rat. Neurotoxicol Teratol [Internet].

[105] Thomas A, Burant A, Bui N, Graham D, Yuva-Paylor LA, Paylor R (2009). Marble burying reflects a repetitive and perseverative behavior more than novelty-induced anxiety. Psychopharmacology [Internet].

[106] Fried PA (1976). Short and long-term effects of pre-natal cannabis inhalation upon rat offspring. Psychopharmacology [Internet].

[107] Abel EL. Effects of delta 9-THC on pregnancy and offspring in rats. Neurobehav Toxicol Teratol [Internet] 1984; 6(1):29–32. Available from: https://www.ncbi.nlm.nih.gov/pubmed/6325967 6325967

[108] Howard DS, Dhanraj DN, Devaiah CG, Lambers DS. Cannabis use based on urine drug screens in pregnancy and its association with infant birth weight. J Addict Med [Internet]. 2019;13(6):436–41. 10.1097/ADM.0000000000000516. 10.1097/ADM.0000000000000516 30908346

[109] Williams CM, Rogers PJ, Kirkham TC (1998). Hyperphagia in pre-fed rats following oral delta9-THC. Physiol Behav [Internet].

[110] Williams CM, Kirkham TC. Reversal of Δ9-THC hyperphagia by SR141716 and naloxone but not dexfenfluramine. Pharmacol Biochem Behav [Internet] 2002;71(1):333–40. Available from: http://www.sciencedirect.com/science/article/pii/S0091305701006943. 10.1016/s0091-3057(01)00694-3 11812541

[111] Farrimond JA, Whalley BJ, Williams CM. A low-Δ9tetrahydrocannabinol cannabis extract induces hyperphagia in rats. Behav Pharmacol [Internet]. 2010 Dec [cited 2020 Jul 22];21(8):769. Available from: https://journals.lww.com/behaviouralpharm/FullText/2010/12000/A_low__9tetrahydrocannabinol_cannabis_extract.10.aspx. 10.1097/FBP.0b013e328340a062 20975531

[112] Gáll Z, Farkas S, Albert Á, Ferencz E, Vancea S, Urkon M (2020). Effects of chronic cannabidiol treatment in the rat chronic unpredictable mild stress model of depression. Biomolecules [Internet].

[113] Harbison RD, Mantilla-Plata B. Prenatal toxicity, maternal distribution and placental transfer of tetrahydrocannabinol. J Pharmacol Exp Ther [Internet]. 1972; 180(2):446–53. Available from: https://www.ncbi.nlm.nih.gov/pubmed/5010682. 5010682

[114] Vardaris RM, Weisz DJ, Fazel A, Rawitch AB (1976). Chronic administration of delta-9-tetrahydrocannabinol to pregnant rats: studies of pup behavior and placental transfer. Pharmacol Biochem Behav [Internet].

[115] Baker T, Datta P, Rewers-Felkins K, Thompson H, Kallem RR, Hale TW (2018). Transfer of inhaled cannabis into human breast milk. Obstet Gynecol [Internet].

[116] Bertrand KA, Hanan NJ, Honerkamp-Smith G, Best BM, Chambers CD (2018). Marijuana use by breastfeeding mothers and cannabinoid concentrations in breast milk. Pediatrics [Internet].

[117] Sempio C, Wymore E, Palmer C, Bunik M, Henthorn TK, Christians U (2020). Detection of Cannabinoids by LC–MS–MS and ELISA in Breast Milk. J Anal Toxicol [Internet].

[118] Feinshtein V, Erez O, Ben-Zvi Z, Eshkoli T, Sheizaf B, Sheiner E, et al. Cannabidiol enhances xenobiotic permeability through the human placental barrier by direct inhibition of breast cancer resistance protein: an ex vivo study. Am J Obstet Gynecol [Internet]. 2013; 209(6):573e1–573e15. 10.1016/j.ajog.2013.08.005. 10.1016/j.ajog.2013.08.005 23933222

[119] Paradisi A, Pasquariello N, Barcaroli D, Maccarrone M (2008). Anandamide regulates keratinocyte differentiation by inducing DNA methylation in a CB1 receptor-dependent manner. J Biol Chem [Internet].

[120] Pucci M, Rapino C, Di Francesco A, Dainese E, D’Addario C, Maccarrone M (2013). Epigenetic control of skin differentiation genes by phytocannabinoids. Br J Pharmacol [Internet].

[121] Eden A, Gaudet F, Waghmare A, Jaenisch R (2003). Chromosomal instability and tumors promoted by DNA hypomethylation. Science [Internet].

[122] Fan G, Beard C, Chen RZ, Csankovszki G, Sun Y, Siniaia M, et al. DNA hypomethylation perturbs the function and survival of CNS neurons in postnatal animals. J Neurosci [Internet]. 2001;21(3):788–97. Available from: https://www.ncbi.nlm.nih.gov/pubmed/11157065. 10.1523/JNEUROSCI.21-03-00788.2001 PMC6762314 11157065

[123] Neidhart M, Rethage J, Kuchen S, Künzler P, Crowl RM, Billingham ME, et al. Retrotransposable L1 elements expressed in rheumatoid arthritis synovial tissue: association with genomic DNA hypomethylation and influence on gene expression. Arthritis Rheum [Internet]. 2000; 43(12):2634–47. 10.1002/1529-0131(200012)43:12<2634::AID-ANR3>3.0.CO;2-1. 10.1002/1529-0131(200012)43:12<2634::AID-ANR3>3.0.CO;2-1 11145021

[124] Howard G, Eiges R, Gaudet F, Jaenisch R, Eden A (2008). Activation and transposition of endogenous retroviral elements in hypomethylation induced tumors in mice. Oncogene [Internet].

[125] Weaver ICG, Meaney MJ, Szyf M (2006). Maternal care effects on the hippocampal transcriptome and anxiety-mediated behaviors in the offspring that are reversible in adulthood. Proc Natl Acad Sci USA [Internet].

[126] Weaver ICG, Cervoni N, Champagne FA, D’Alessio AC, Sharma S, Seckl JR (2004). Epigenetic programming by maternal behavior. Nat Neurosci [Internet].

[127] Cameron NM, Shahrokh D, Del Corpo A, Dhir SK, Szyf M, Champagne FA (2008). Epigenetic programming of phenotypic variations in reproductive strategies in the rat through maternal care. J Neuroendocrinol [Internet].

[128] Szyf M, Weaver I, Meaney M (2007). Maternal care, the epigenome and phenotypic differences in behavior. Reprod Toxicol [Internet].

[129] McGowan PO, Suderman M, Sasaki A, Huang TCT, Hallett M, Meaney MJ (2011). Broad epigenetic signature of maternal care in the brain of adult rats. PLoS ONE [Internet].

[130] Meaney MJ (2001). Maternal care, gene expression, and the transmission of individual differences in stress reactivity across generations. Annu Rev Neurosci [Internet].

[131] Liu D, Diorio J, Tannenbaum B, Caldji C, Francis D, Freedman A (1997). Maternal care, hippocampal glucocorticoid receptors, and hypothalamic-pituitary-adrenal responses to stress. Science [Internet].

[132] Costa HHV, Vilela FC, Giusti-Paiva A (2013). Continuous central infusion of cannabinoid receptor agonist WIN 55212-2 decreases maternal care in lactating rats: consequences for fear conditioning in adulthood males. Behav Brain Res [Internet].

[133] Colizzi M, Bhattacharyya S (2017). Does cannabis composition matter? Differential effects of delta-9-tetrahydrocannabinol and cannabidiol on human cognition. Curr Addict Rep [Internet].

[134] Fagherazzi EV, Garcia VA, Maurmann N, Bervanger T, Halmenschlager LH, Busato SB (2012). Memory-rescuing effects of cannabidiol in an animal model of cognitive impairment relevant to neurodegenerative disorders. Psychopharmacology [Internet].

[135] Lee JLC, Bertoglio LJ, Guimarães FS, Stevenson CW (2017). Cannabidiol regulation of emotion and emotional memory processing: relevance for treating anxiety-related and substance abuse disorders. Br J Pharmacol [Internet].

[136] Norris C, Loureiro M, Kramar C, Zunder J, Renard J, Rushlow W (2016). Cannabidiol modulates fear memory formation through interactions with serotonergic transmission in the mesolimbic system. Neuropsychopharmacology [Internet].

[137] Crippa JAS, Derenusson GN, Ferrari TB, Wichert-Ana L, Duran FLS, Martin-Santos R (2011). Neural basis of anxiolytic effects of cannabidiol (CBD) in generalized social anxiety disorder: a preliminary report. J Psychopharmacol [Internet].

[138] Zuardi AW, Crippa JAS, Hallak JEC, Moreira FA, Guimarães FS (2006). Cannabidiol, a Cannabis sativa constituent, as an antipsychotic drug. Braz J Med Biol Res [Internet].

[139] Morgan CJA, Freeman TP, Hindocha C, Schafer G, Gardner C, Curran HV (2018). Individual and combined effects of acute delta-9-tetrahydrocannabinol and cannabidiol on psychotomimetic symptoms and memory function. Transl Psychiatry [Internet].

[140] Luján MÁ, Valverde O (2020). The pro-neurogenic effects of cannabidiol and its potential therapeutic implications in psychiatric disorders. Front Behav Neurosci [Internet].

[141] Olson AK, Eadie BD, Ernst C, Christie BR (2006). Environmental enrichment and voluntary exercise massively increase neurogenesis in the adult hippocampus via dissociable pathways. Hippocampus [Internet].

[142] Fabel K, Wolf SA, Ehninger D, Babu H, Leal-Galicia P, Kempermann G (2009). Additive effects of physical exercise and environmental enrichment on adult hippocampal neurogenesis in mice. Front Neurosci [Internet].

[143] Park H-S, Kim T-W, Park S-S, Lee S-J. Swimming exercise ameliorates mood disorder and memory impairment by enhancing neurogenesis, serotonin expression, and inhibiting apoptosis in social isolation rats during adolescence. J Exerc Rehabil [Internet]. 2020; 16(2):132–40. 10.12965/jer.2040216.108. 10.12965/jer.2040216.108 PMC7248435 32509697

[144] Snyder JS, Soumier A, Brewer M, Pickel J, Cameron HA (2011). Adult hippocampal neurogenesis buffers stress responses and depressive behaviour. Nature [Internet].

[145] Sahay A, Hen R (2007). Adult hippocampal neurogenesis in depression. Nat Neurosci [Internet].

[146] Wegiel J, Kuchna I, Nowicki K, Imaki H, Wegiel J, Marchi E (2010). The neuropathology of autism: defects of neurogenesis and neuronal migration, and dysplastic changes. Acta Neuropathol [Internet].

[147] Gilbert J, Man H-Y (2017). Fundamental elements in autism: from neurogenesis and neurite growth to synaptic plasticity. Front Cell Neurosci [Internet].

[148] Kaushik G, Zarbalis KS (2016). Prenatal neurogenesis in autism spectrum disorders. Front Chem [Internet].

