# Supplementary figures and images for: Developmental cannabidiol exposure increases anxiety and modifies genome-wide brain DNA methylation in adult female mice
Source: Clin Epigenetics. 2021 Jan 6;13:4. doi: 10.1186/s13148-020-00993-4 (PMC7789000; doi:10.1186/s13148-020-00993-4)

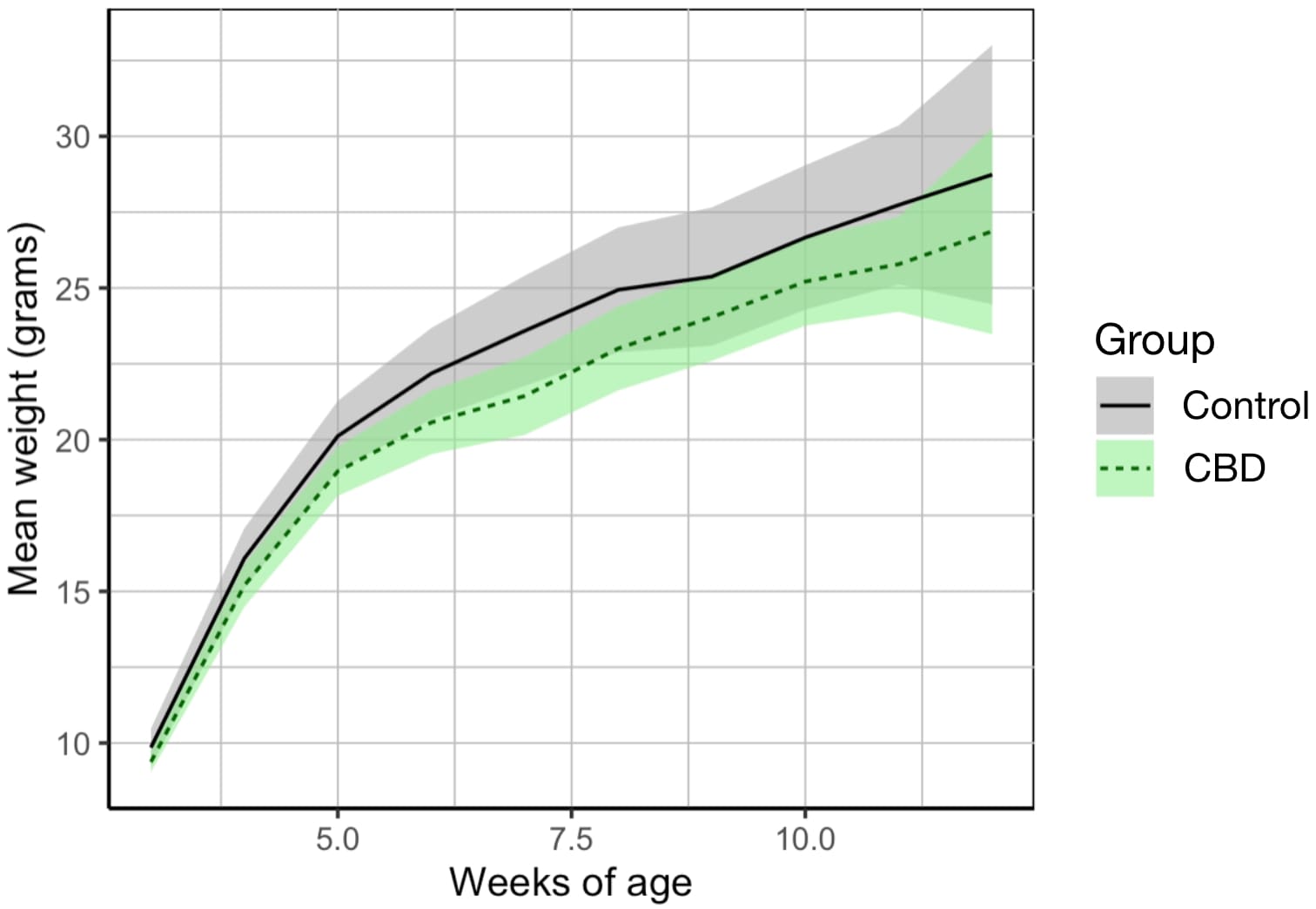

Supplement: Supplementary file 2 — Additional file 2: Body weight trends for wild-type a/a F1 offspring of both sexes from weaning through study completion (12 weeks). Shaded areas represent 95% confidence intervals. Weights did not differ significantly between groups at any time point by ANOVA (p = 0.995). [file 13148_2020_993_MOESM2_ESM.jpg]

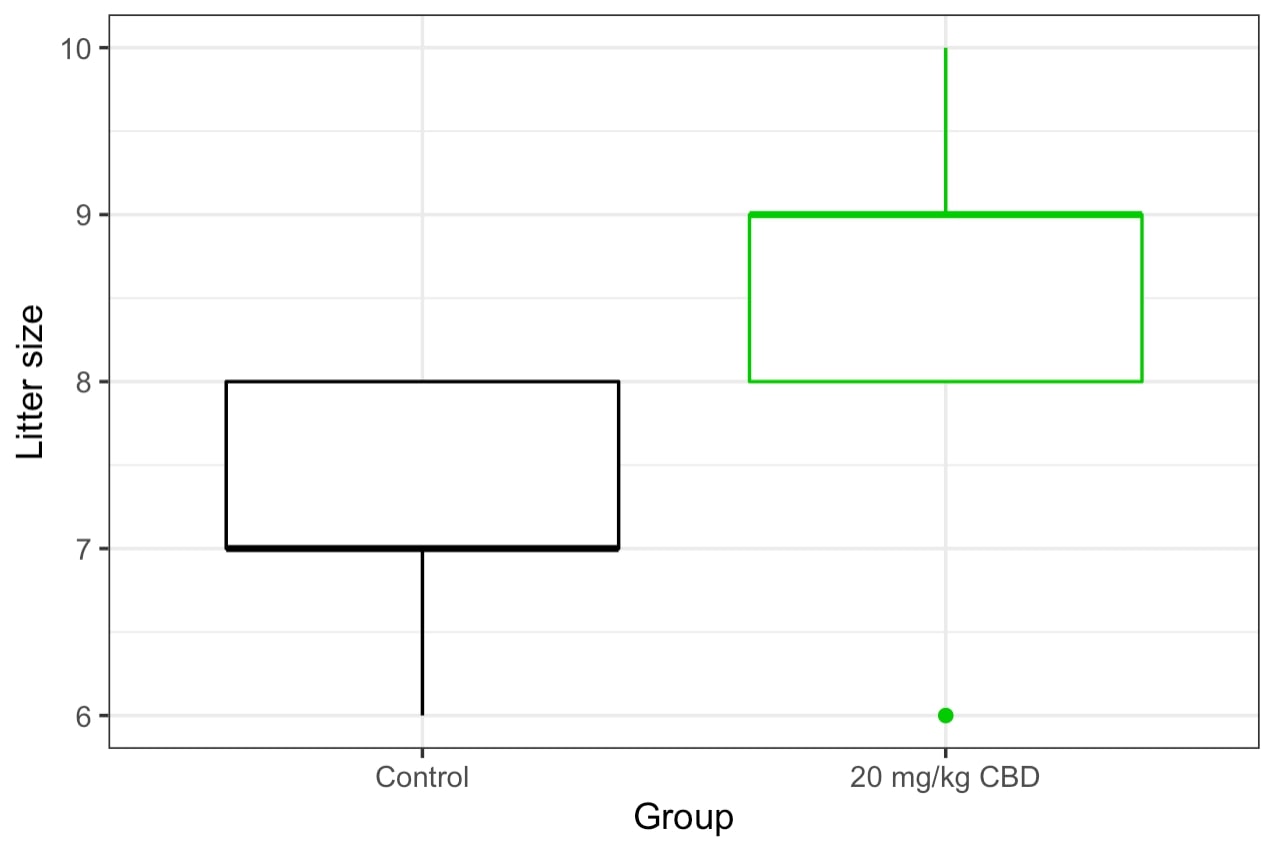

Supplement: Supplementary file 3 — Additional file 3: Litter size for F1 pups differed significantly between groups with CBD-exposed litters containing 1.25 more pups on average in comparison to control litters (p = 0.0134). [file 13148_2020_993_MOESM3_ESM.jpg]

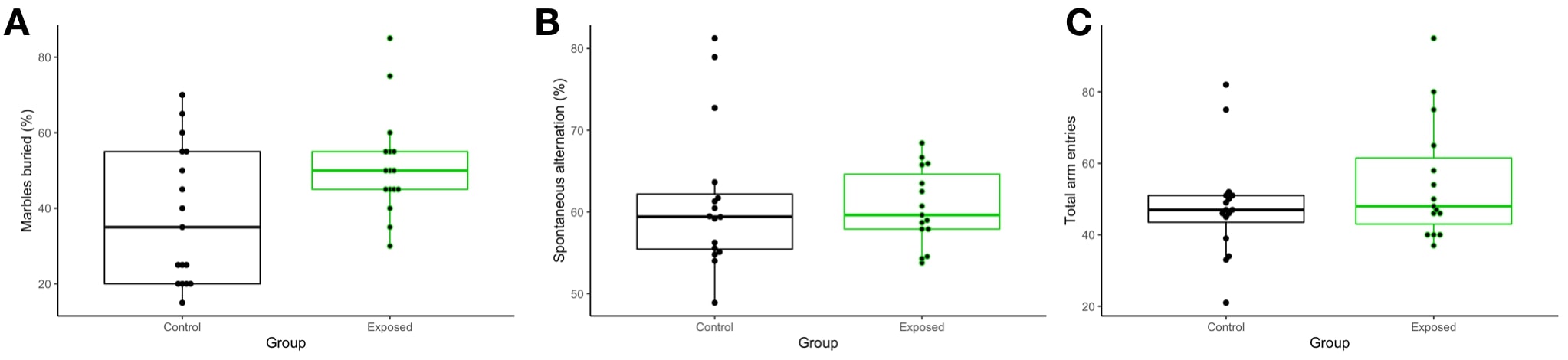

Supplement: Supplementary file 4 — Additional file 4: Scores for wild-type a/a F1 young adult mice in (a) marble burying, a measure of anxiety and (b) Y-maze spontaneous alternation and (c) Y-maze arm entries, measures of spatial memory, did not differ significantly between CBD-exposed and control groups when results from both sexes were combined. [file 13148_2020_993_MOESM4_ESM.jpg]

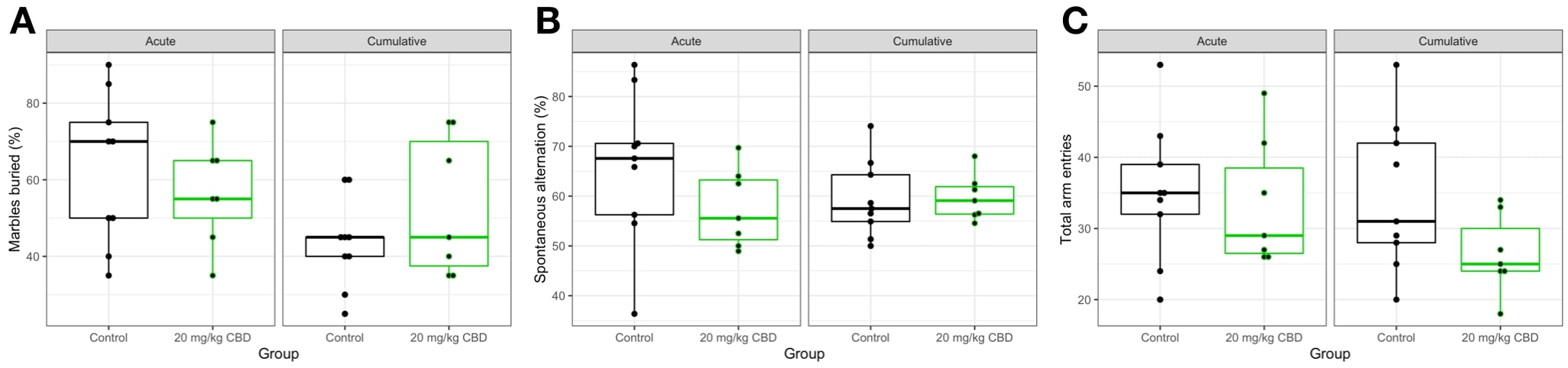

Supplement: Supplementary file 5 — Additional file 5: Scores for wild-type a/a F0 adult female mice in (a) marble burying, a measure of anxiety and (b) Y-maze spontaneous alternation and (c) Y-maze arm entries, measures of spatial memory and locomotion, did not differ significantly between animals receiving 20 mg/kg CBD daily for 9 weeks and controls for either acute (test performed near CBD Cmax) or cumulative (test performed 24 hours after last dose) runs. Likewise, paired comparisons for acute vs. cumulative scores were not statistically significant. [file 13148_2020_993_MOESM5_ESM.jpg]

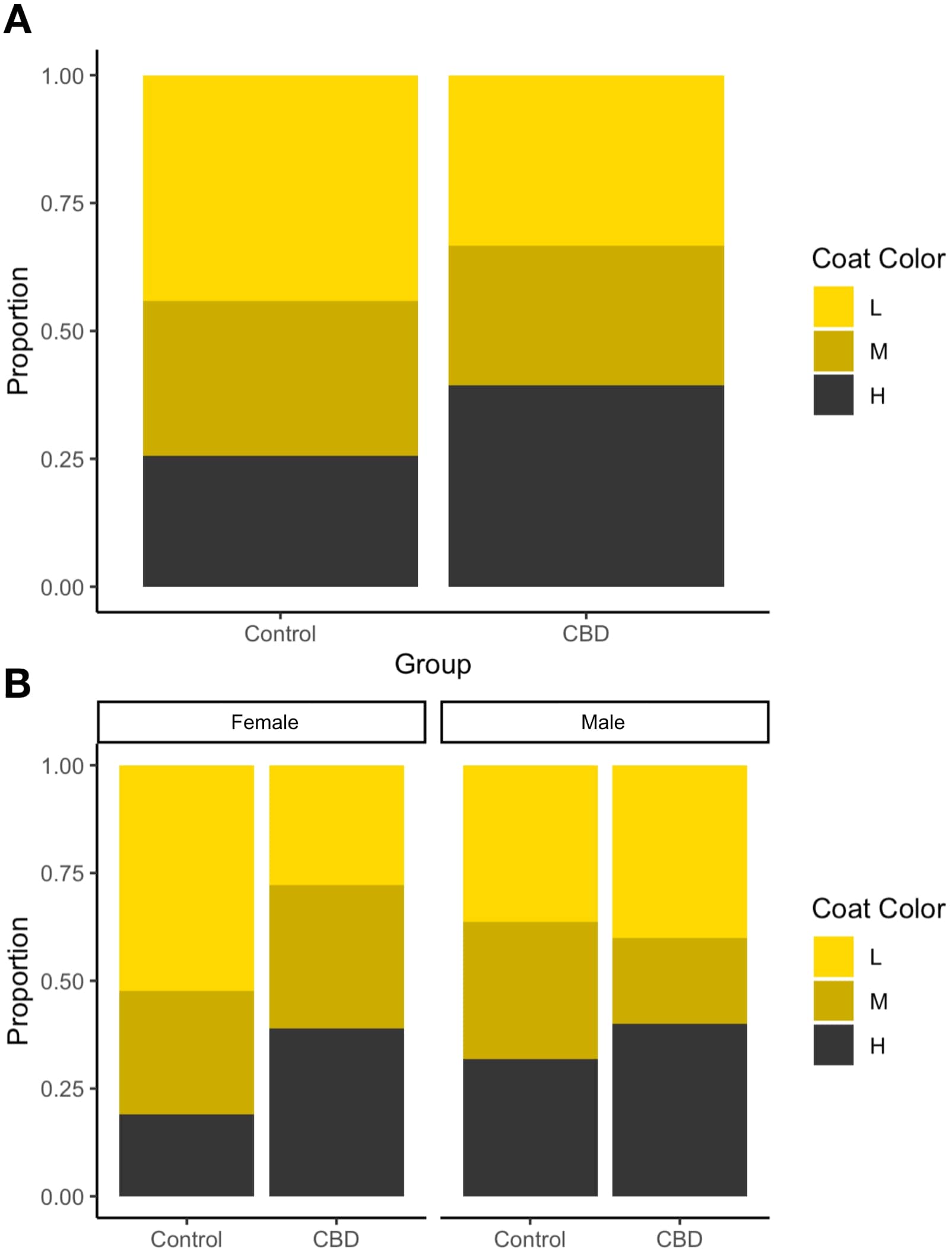

Supplement: Supplementary file 6 — Additional file 6: Measurements of F1 Avy/a offspring coat color on a three category scale (L: low methylation/yellow; M: medium methylation/mottled; H: high methylation/pseudoagouti) revealed a lack of statistically significant differences between CBD-exposed and control groups when assessed (a) as a whole and (b) stratified by sex. [file 13148_2020_993_MOESM6_ESM.jpg]

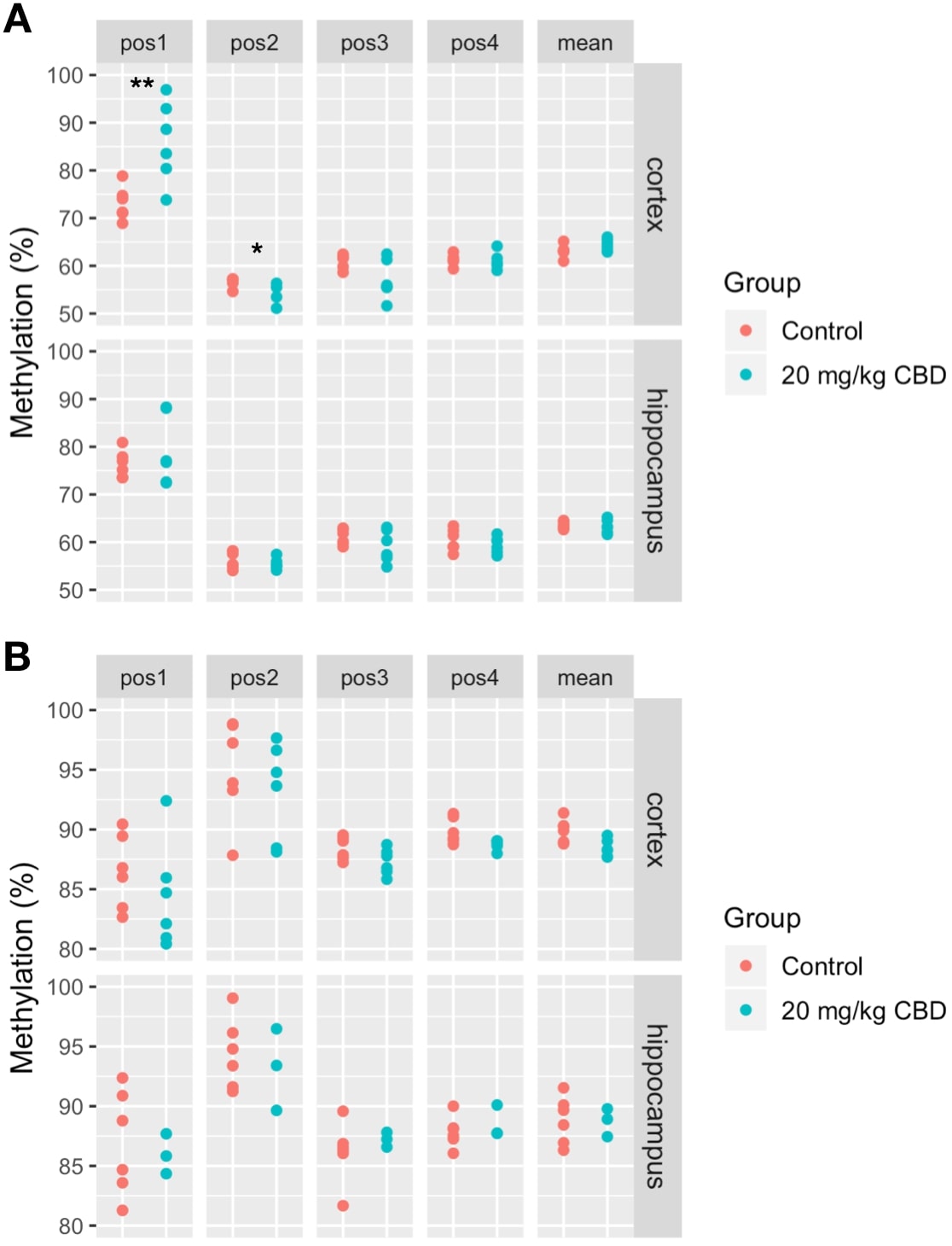

Supplement: Supplementary file 8 — Additional file 8: Methylation values (four CpG positions and mean) for CpGs lying in (a) mLINE1 and (b) IAP retrotransposons in F0 female cerebral cortex and hippocampus. Significant hypermethylation was identified at mLINE1 position 1 and hypomethylation at mLINE1 position 2 in CBD-exposed tissues, both in cerebral cortex only. Other mLINE1 positions, mean methylation, and IAP positions and mean were not found to be significantly different between groups. Each point represents an individual animal. * = p < 0.05, ** = p < 0.01. [file 13148_2020_993_MOESM8_ESM.jpg]
